# Impact of the citizen science project COLLECT on ocean literacy and well-being within a north/west African and south-east Asian context

**DOI:** 10.3389/fpsyg.2023.1130596

**Published:** 2023-06-14

**Authors:** Marine I. Severin, Lazare Kouame Akpetou, Pavanee Annasawmy, Francis Emile Asuquo, Fiona Beckman, Mostapha Benomar, Annette Jaya-Ram, Mohammed Malouli, Jan Mees, Ivanice Monteiro, Joey Ndwiga, Péricles Neves Silva, Olubunmi Ayoola Nubi, Yee Kwang Sim, Zacharie Sohou, Aileen Tan Shau-Hwai, Sau Pinn Woo, Soukaina Zizah, Ann Buysse, Filip Raes, Lilian A. Krug, Sophie Seeyave, Gert Everaert, Edem Mahu, Ana I. Catarino

**Affiliations:** ^1^Flanders Marine Institute (VLIZ), Oostende, Belgium; ^2^Department of Experimental Clinical and Health Psychology, Ghent University, Ghent, Belgium; ^3^Centre for the Psychology of Learning and Experimental Psychopathology, KU Leuven, Leuven, Belgium; ^4^Centre Universitaire de Recherche et d’Application en Télédétection (CURAT), Université Félix Houphouët-Boigny, Abidjan, Côte d’Ivoire; ^5^Université de Bretagne Occidentale, Plouzané, France; ^6^Marine Atmosphere and Coastal Ocean Research Network (MACORN), UNICAL, Faculty of Oceanography, University of Calabar, Calabar, Nigeria; ^7^Partnership for Observation of the Global Ocean (POGO), Plymouth, United Kingdom; ^8^Institut National de Recherche Halieutique (INRH), Casablanca, Morocco; ^9^Centre for Marine and Coastal Studies (CEMACS), Universiti Sains Malaysia, Pulau Pinang, Malaysia; ^10^Marine Biology Research Group, Faculty of Sciences, Ghent University, Ghent, Belgium; ^11^Ocean Science Centre Mindelo, Instituto do Mar (IMar), Mindelo, Cape Verde; ^12^Nigerian Institute for Oceanography and Marine Research (NIOMR), Lagos, Nigeria; ^13^Institut de Recherches Halieutiques et Océanologiques du Benin (IRHOB), Cotonou, Benin; ^14^Centre for Marine and Environmental Research (CIMA), University of Algarve, Faro, Portugal; ^15^Department of Marine and Fisheries Sciences, University of Ghana, Accra, Ghana

**Keywords:** plastic pollution, beach sampling, citizen science, ocean literacy, pro-environmental intentions, well-being

## Abstract

Plastic pollution is both a societal and environmental problem and citizen science has shown to be a useful tool to engage both the public and professionals in addressing it. However, knowledge on the educational and behavioral impacts of citizen science projects focusing on marine litter remains limited. Our preregistered study investigates the impact of the citizen science project *Citizen Observation of Local Litter in coastal ECosysTems* (COLLECT) on the participants’ ocean literacy, pro-environmental intentions and attitudes, well-being, and nature connectedness, using a pretest-posttest design. A total of 410 secondary school students from seven countries, in Africa (Benin, Cabo Verde, Côte d’Ivoire, Ghana, Morocco, Nigeria) and Asia (Malaysia) were trained to sample plastics on sandy beaches and to analyze their collection in the classroom. Non-parametric statistical tests (*n* = 239 matched participants) demonstrate that the COLLECT project positively impacted ocean literacy (i.e., awareness and knowledge of marine litter, self-reported litter-reducing behaviors, attitudes towards beach litter removal). The COLLECT project also led to higher pro-environmental behavioral intentions for students in Benin and Ghana (implying a positive spillover effect) and higher well-being and nature connectedness for students in Benin. Results are interpreted in consideration of a high baseline in awareness and attitudes towards marine litter, a low internal consistency of pro-environmental attitudes, the cultural context of the participating countries, and the unique settings of the project’s implementation. Our study highlights the benefits and challenges of understanding how citizen science impacts the perceptions and behaviors towards marine litter in youth from the respective regions.

## 1. Introduction

The accumulation of plastic litter in the environment, and its impact on ecosystem services (e.g., fisheries, tourism, maritime transportation) and human well-being is a concern of public interest. Economic activities of local populations, such as fisheries or seafood sales, can be affected indirectly by potential effects of litter on fish stocks (e.g., due to ghost fishing gear) or by consumers perception of shellfish as unsafe items for purchasing, due to the presence of microplastics (Van Cauwenberghe and Janssen, 2014). Furthermore, there is growing evidence that direct and/or indirect exposure to coastal environments can benefit various aspects of well-being (e.g., social relationships, restoration from stress and attentional fatigue, positive emotions; Hooyberg et al., 2020; White et al., 2020; Severin et al., 2021b, 2022). However, the presence of marine litter can negatively impact these benefits by reducing the coast’s restorative and recreational qualities and inducing negative moods (Wyles et al., 2016; De Veer et al., 2022). Marine litter can also disrupt the aesthetic experience of the coast by diminishing its scenic quality (Rangel-Buitrago et al., 2018), thereby reducing the chances of people spending time in (littered) coasts (Tudor and Williams, 2006) and negatively affecting the tourism industry (Williams et al., 2016; Krelling et al., 2017). In terms of risks of marine litter on human health, a recent report by the World Health Organization (WHO) concludes that although the evidence remains insufficient, a reduction in exposure would greatly benefit humans and the environment (World Health Organization, 2022).

To tackle the problem of plastic pollution, action needs to be taken at both collective and individual levels, and citizen science can be a useful tool to engage both the general public and STEM (science, technology, engineering, and mathematics) professionals. Global, national and local authorities recognize the urgency to mitigate this issue and various community initiatives frequently take place aiming at protecting natural and recreational areas, such as beach “clean-up” activities, where plastic items are manually collected (Jorgensen et al., 2021). Examples of such beach clean-ups include the Ocean Conservancy’s International Coastal Cleanup that engages volunteers and organizations to remove debris from beaches and waterways worldwide (Ocean Conservancy, 2022), and “Clean up the Med,” a nationwide campaign of voluntary beach clean-ups in Greece (Kordella et al., 2013). Citizen science goes a step beyond by actively involving citizens in the scientific research process, which can promote action by first, addressing data gaps in marine litter distribution and abundance to develop effective mitigation measures, and second, increasing public awareness of plastic pollution and encouraging individual action to reduce plastic littering (Thiel et al., 2011; Hidalgo-Ruz and Thiel, 2013).

The individual level, with a focus on the citizen participation, is however often neglected in the literature. Despite the growing number of citizen science projects focused on plastic pollution (Rambonnet et al., 2019), only a small percentage evaluate their effectiveness in impacting awareness and sustainable action on their participants (Severin et al., 2023). Including this evaluation is nonetheless essential to understand each project’s benefits and challenges presented to the public, thereby maximizing its full potential to address plastic pollution on a local and global scale (Kelly et al., 2022). Moreover, as stated in Pahl and Wyles (2017), understanding the perceptions, decisions, and actions of humans is a central ingredient in undertaking the issue of plastic pollution. Additionally, there is a strong underrepresentation of African countries within citizen science projects, accompanied with a lack of socio-economic and socio-demographic diversity (Kawabe et al., 2022; Severin et al., 2023). In this paper, we investigate the impact of the *Citizen Observation of Local Litter in coastal ECosysTems* (COLLECT) project on the participants’ ocean literacy, pro-environmental intentions and attitudes, and well-being. Ocean literacy was conceptualized as awareness and knowledge of marine plastic litter, self-reported litter-reducing behaviors, and attitudes towards beach litter removal.

### 1.1. The COLLECT project

The COLLECT project (2021–2022) was a citizen science initiative with the aim of obtaining data on the abundance and distribution of coastal plastic litter in seven countries in North and West Africa (Benin, Cabo Verde, Côte d’Ivoire, Ghana, Morocco, Nigeria) and South-East Asia (Malaysia). The project consisted of training students from secondary schools (11–18 years old) in sampling and analyzing macro-, meso- and microplastic in sandy beaches. Students went to the beach during two different seasons (wet-autumn and dry-spring) and sampled plastic following a standard operating procedure (SOP). They then analyzed the samples in the classroom. Prior to the field activities, the students were given an information session which consisted of presenting the project and providing background information on plastic pollution and guidelines for field sampling. COLLECT’s methodology followed best practices of citizen science initiatives on plastic litter assessments in aquatic areas (Rambonnet et al., 2019; Barnardo and Ribbink, 2020; De Rijck et al., 2020). The project was overseen by the Partnership for Global Ocean Observation (POGO),^1^ a well-established network of oceanographic research institutes that collaborate to promote and execute global ocean observations *via* innovation, capacity building, outreach, and advocacy (Miloslavich et al., 2019; POGO, 2021). In each country, the project was undertaken by local researchers who were affiliated within member institutions of the POGO. More detailed information regarding the full methodology of the project can be found in Catarino et al. (2023).

### 1.2. Prevalence and impacts of litter in the participating countries

Currently, littoral countries in Africa are leaders in the world’s urbanization rates (Saghir and Santoro, 2018), and there are concerns over a parallel drastic increase in the production and release of municipal solid waste in these areas (Yoshida, 2018) potentially accumulating on the coast (Ryan, 2020). In Africa, the total mismanaged plastic waste in 2010 was estimated to be 4.4 million metric tons, and is estimated to reach 10.5 million metric tons in 2025 (Jambeck et al., 2018). Strict policies on plastic imports and on the ban on single-use plastic items have demonstrated efficiency in the reduction of plastic consumption, such as in the case of Rwanda (Babayemi et al., 2019), however the effectiveness of such measures without a complete life-cycle approach is not yet clarified (The Economist, 2019). For example, according to the LitterBase (Bergmann et al., 2017),^2^ marine litter data is scarce for most of the coastal areas in Africa. The situation is similar in Malaysia, as it is one of the leading countries regarding the generation of plastic waste as well as the consumption of single-use plastics (Chen et al., 2021). More than 0.94 million metric tons of mismanaged plastic waste is generated per year, of which an estimated 0.14–0.37 million metric tons consist of plastic marine debris (Jambeck et al., 2015). Key components constraining waste management include an inconsistent application of policy initiatives and a lack of public awareness and interest in household recycling (Chen et al., 2021). Marine litter data also remains lacking in Malaysia (Fauziah et al., 2021). The project COLLECT aimed to address this data gap and provide baseline data, crucial to propose solutions and mitigation measures for the reduction of coastal debris impacts in emerging economies and vulnerable coastal communities.

According to a review by Arabi et al. (2023), there is limited information regarding the social, economic, and health impacts of marine litter in the participating countries, with most of the literature focusing on South Africa. Arabi et al. (2023) demonstrate that the presence of marine litter reduces the aesthetic value of popular tourist beaches in Benin and Côte d’Ivoire, and that unclean beaches were one of the main concerns of coastal residents in Ghana (Van Dyck et al., 2016). In Nigeria, perceived major impacts of marine litter also include aesthetic impairment, as well as health issues and economic downscale (Nubi et al., 2019). Furthermore, marine litter is shown to directly affect the tourism industry, particularly in the African Small Island Developing States (SIDS; including Cabo Verde). SIDS tourism depends on “beautiful,” clean beaches, but are the most disproportionately affected by marine litter, and therefore necessitate continuous cleaning of the beaches at a high economic cost (Arabi et al., 2023). In 2015, the economic cost of damage from marine litter to fisheries, aquaculture, marine transport, shipbuilding and tourism in the APEC (Asia-Pacific Economic Cooperation) region, including Malaysia, was estimated at 11.2 billon USD (Mcllgorm et al., 2020). Finally, the participating countries are also faced with the health risks presented by marine litter through the consumption of seafood containing microplastics, as shown in Malaysia (Karbalaei et al., 2019), and potential injuries due to sharp plastic fragments or discarded fishing nets in the water (Van Dyck et al., 2016; Arabi et al., 2023).

### 1.3. The impacts of citizen science on participants

The goal of increasing ocean literacy in the global population is to enable a positive behavior change towards the ocean and its resources, according to the revised roadmap for the United Nations (UN) Decade of Ocean Science for Sustainable Development (Intergovernmental Oceanographic Commission, 2018). Ocean literacy has been broadly defined up to now as “an understanding of the ocean’s influence on you and your influence on the ocean” (Schoedinger et al., 2005, p. 1). There is however a growing consensus that ocean literacy should not only pertain to understanding of ocean-related topics, but should further include attitudes, behaviors, and level of connectedness to the ocean (Kelly et al., 2022). A recent study demonstrated that ocean literacy consisted of six sub-dimensions: knowledge of ocean-related topics, personal interest in ocean-related aspects, ocean stewardship, ocean as an economic resource, ocean-friendly behavior, and willingness to act responsibly towards the ocean (Paredes-Coral et al., 2022). One conceptual approach that can be utilized to achieve positive behavior change towards the ocean is the Theory of Change model (Connell and Kubisch, 1998). This approach consists of first determining an end objective and then identifying the different steps needed to be reached to obtain this objective. Ashley et al. (2019) demonstrated how the theory of change model can be applied to evaluate ocean literacy initiatives targeting specific behavior change. The identified steps predicting the targeted behavior change were: awareness and knowledge of the issue, attitudes (concern) and self-efficacy (belief that one’s own actions will be beneficial) towards the issue, and interpersonal communication about the issue with friends and family. The ocean literacy initiatives were found to positively impact every step of the theory of change model, as well as the intended behavior change. The study thereby displayed the effectiveness of the included predictors of behavior change and their relevance within the ocean literacy framework (Ashley et al., 2019).

Citizen science projects related to marine litter are found to positively impact components of ocean literacy (Hartley et al., 2015; Yeo et al., 2015; Wyles et al., 2017; Locritani et al., 2019; Rayon-Viña et al., 2019; Wichmann et al., 2022). Hartley et al. (2015) assessed the effect of an educational intervention on young school children and demonstrated that following the intervention, children reported more concern towards marine litter, had better knowledge of its causes and consequences, and engaged in more sustainable actions aimed at reducing marine litter. Locritani et al. (2019) used the same questionnaire as Hartley et al. (2015), with adolescents, and found similar results with exception to lower variation in problem awareness and perceived negative impacts of marine litter. This was likely to be related to a higher baseline awareness and knowledge of plastic pollution. Similarly, a study by Wichmann et al. (2022) reported a high baseline problem perception and involvement towards marine plastic litter in Chilean school children and did not find any significant effects of the citizen science initiative on their participants, except for an increase in perceived negative impacts. In addition to the high baseline causing little room for improvement, the authors suggested that citizen science alone is not enough to generate pro-environmental behavior change, but would benefit from the inclusion of educational modules that teach participants strategies and skills to empower them for environmental protection (Wichmann et al., 2022). More research is therefore needed to better understand how citizen science can be optimized in its potential to impact ocean literacy.

In addition to benefiting ocean literacy, participating in citizen science initiatives may lead to a positive “spillover effect” (i.e., an indirect side effect of an intervention), in which engaging in one pro-environmental behavior might encourage further engagement in other pro-environmental behaviors (Thøgersen and Ölander, 2003). This was demonstrated in the study by Wyles et al. (2017), in which participating in a beach clean-up led to increased intentions to engage in other, more generic, pro-environmental behaviors. Indeed, a recent Bayesian meta-analysis based on environmental interventions found small positive spillover effects for sustainable intentions, however no spillover effects were found for sustainable behaviors (Geiger et al., 2021). Although spillover effects are generally considered in terms of intentions and behaviors, we would like to explore whether they could apply in terms of attitudes as well. More specifically, if citizen science can impact attitudes regarding a particular issue (e.g., marine litter), perhaps this can also increase pro-environmental attitudes in general.

It has been further demonstrated that citizen science initiatives positively impact well-being and nature connectedness (Koss and Kingsley, 2010; Yeo et al., 2015; Wyles et al., 2017). In the study by Wyles et al. (2017), after participating in a beach clean-up, students reported feeling a more positive mood and higher eudaimonic well-being. In this context, eudaimonic well-being was referred to the meaningfulness of the activity and whether it was in line with one’s personal values. Moreover, Koss and Kingsley (2010) investigated the well-being of volunteers of a marine monitoring program and found that volunteering created personal satisfaction, feelings of enjoyment, and feeling pride in themselves. It is important to note however that the presence of litter in coastal environments can diminish the restorative qualities of the coast and induce a negative mood (Wyles et al., 2016). Nonetheless, Wyles et al. (2017) speculate that although the littered environment is less restorative, the activity (e.g., beach cleaning) itself may counteract the potential negative effects by benefiting hedonic and eudaimonic well-being. With regards to nature connectedness, the volunteers in Koss and Kingsley (2010) reported an increased sense of connection to nature, which was linked with a desire to protect the coastal environment. This is in line with findings of a positive relationship between connectedness to nature and pro-environmental behavior (Martin et al., 2020). Additionally, Wyles et al. (2019) demonstrate that exposure to coastal environments was associated with higher nature connectedness. We can thus speculate that interacting with the coast can boost connectedness with nature, which in turn can facilitate positive behavior change.

### 1.4. Present study

The aim of the present study was to assess the impact of the citizen science intervention in the project COLLECT (Citizen Observation of Local Litter in Coastal ECosysTems), by evaluating shifts in participants’ ocean literacy, pro-environmental intentions and attitudes, and well-being. To achieve this, students were asked to complete a survey both before (pre-survey) and after (post-survey) the project activities, using a within-subject design. Ocean literacy was conceptualized based on previous studies evaluating citizen science initiatives (Hartley et al., 2015; Wyles et al., 2017; Locritani et al., 2019; Lucrezi and Digun-Aweto, 2020) and the predictors of behavior change from the Theory of Change model used in Ashley et al. (2019). More specifically, to assess ocean literacy we measured awareness and knowledge of marine plastic litter, self-reported litter-reducing behaviors, and attitudes towards beach litter removal. Pro-environmental behavioral intentions and attitudes were included to assess potential spillover effects. Finally, we evaluated hedonic and eudaimonic well-being, as well as nature connectedness.

## 2. Methods

The current study’s experimental design and data analysis plan were preregistered in the Open Science Framework (OSF) registry before data collection (Severin et al., 2021a).^3^

### 2.1. Participants

A total of 410 secondary school students from seven different countries took part in the surveys evaluating the impact of COLLECT. About half of the participants identified as female (49.5%), and 43% identified as male (7.5% gave no response). The mean age was 15.8 years old (*SD* = 2.19), with the youngest participant being 11 years old and the oldest being 22 years old. The majority of participants (72.4%) were between 14 and 18 years old. Table 1 depicts the distribution of participants according to country, along with socio-demographic characteristics. There was an important gap in sample size between the countries as certain countries (e.g., Cabo Verde) typically have very low attendance of secondary school students compared to other countries which usually have 50 students per class (e.g., Nigeria). A total of 171 students could not be matched due to missing pre- (*n* = 72) or post-surveys (*n* = 99). Matched pre- and post-surveys that were therefore included in the analysis represent 58% of the total sample size (*n* = 239). Various factors can explain this attrition such as students not attending class at both time points, mistyped identification codes, or schools not being able to finish the sampling activities due to time constraints.

**Table 1:** Participant characteristics.

Country (total sample; matched pre- and post-surveys)	School	Age distribution	Gender distribution^a^
Benin (*n* = 66; *n* = 65)	Lycée Technique Coulibaly	*M* = 17.6, *SD* = 1.91, *min* = 12, *max* = 22	Female = 39.4% Male = 60.6%
Cabo Verde (*n* = 29; *n* = 16)	Escola Salesiana de Artes e Ofícios	*M* = 16.8, *SD* = 1.05, *min* = 16, *max* = 20	Female = 79.3% Male = 13.8%
	Liceu Ludgero Lima		
Côte d’Ivoire (*n* = 29; *n* = 11)	Collège les Oliviers de Port Bouet	*M* = 17.4, *SD* = 1.57, *min* = 14, *max* = 21	Female = 58.6% Male = 34.5%
Ghana (*n* = 67; *n* = 34)	O’Reilly Senior High School	*M* = 17, *SD* = 1.18, *min* = 15, *max* = 21	Female = 40.3% Male = 55.2%
Malaysia (*n* = 64; *n* = 37)	Prince of Wales Island International School	*M* = 14.6, *SD* = 1.54, *min* = 12, *max* = 18	Female = 45.3% Male = 54.7%
Morocco (*n* = 59; *n* = 1)	Lycée des Sportifs	*M* = 15.8, *SD* = 2.06, *min* = 12, *max* = 21	Female = 40.7% Male = 22%
	École IBN Batouta		
Nigeria (*n* = 96; *n* = 75)	Riverside College	*M* = 14, *SD* = 1.81, *min* = 11, *max* = 21	Female = 59.4% Male = 38.5%
	University of Calabar International Secondary School		

^a^Remaining percentage (to total 100%) corresponds to missing data.

### 2.2. Measures

The pre- and post-assessment survey contained a series of questions divided into three main topics: awareness and knowledge of marine litter, attitudes and behaviors towards marine litter and the environment, and well-being, including nature connectedness. Questions regarding socio-demographics (i.e., gender, age) and visit frequency to the beach were also included. Additionally, the post-survey contained questions measuring satisfaction towards the COLLECT project. The survey was written in English (applied in Ghana, Malaysia, and Nigeria) and then translated to French (applied in Benin, Côte d’Ivoire, and Morocco) and Portuguese (applied in Cabo Verde). The survey language was in accordance with the school language of each country, with exception to the Moroccan schools who employed Spanish, however the students also spoke French. All surveys are open access.^4^ When possible, we used translated scales that had already been validated in empirical studies. All other translated items or scales were reviewed by two independent native speakers. To ensure that the survey questions were not too difficult to comprehend or answer, we piloted the survey with the students from Nigeria, from the University of Calabar International Secondary School. The students could indicate when they did not understand the question and could give general feedback. The schools conducted the survey in paper format, with the exception of the school in Malaysia that used an online version of the survey on the Limesurvey platform (Limesurvey GmbH).^5^

#### 2.2.1. Awareness and knowledge of marine litter

Awareness of marine litter was represented by participants’ problem awareness and concern regarding marine litter, and their perceived impacts and causes of marine litter. Questions were adjusted from Hartley et al. (2015) and Locritani et al. (2019) and were reworded in the format of five-point Likert agreement scales to create consistency in response options throughout the survey. Problem awareness of marine litter was measured with the statement “Litter on the beach and in the sea is a problem.” Two extra items measuring awareness were included: “litter on the beach and in the sea is a future environmental threat rather than a present one” (based on Hartley et al., 2018) and “litter on the beach and in the sea in our country is more problematic than in other neighboring countries.” Concern towards marine litter was measured with the statement “I am worried about the problems that litter on the beach and in the sea might cause.” Participants then rated their agreement to whether impacts of marine litter on marine wildlife, tourism, human health, fishing activities, and the appearance of the coast were “bad.” Finally, participants rated their agreement to whether marine litter was due to people dropping litter on the beach, not enough bins, businesses and fishing activities, too much packaging that is difficult to recycle, and rivers discharging waste into the sea.

Knowledge of marine litter was measured by asking participants the estimated degradation time of plastic bottles, similar to Hartley et al. (2015). Response options were less than 1 year, 1–10 years, 10–50 years, 50–100 years, or more than 100 years. Ten to fifty years and 50–100 years were considered as correct responses (Edge et al., 1991; Allen et al., 1994). We also included four multiple choice questions to assess perceived salience of marine plastic. Three questions were based on those used in Wyles et al. (2017), i.e., “what do you think was the most common type of litter found on the coastline near you in 2020?,” “over the last 10 years, plastic bottles found on the coastline near you have increased by…,” and “on average in 2020, how many pieces of litter were found per meter on the coastline near you?” The fourth question consisted of asking participants what percentage of marine litter they thought was plastic, as done in Hartley et al. (2015), with response options being 0–10%, 10–25%, 25–50%, 50–75%, or 75–100%.

#### 2.2.2. Attitudes and behaviors towards marine litter and the environment

Attitudes towards marine litter were specifically targeted towards beach litter removal and were measured with items taken from Lucrezi and Digun-Aweto (2020), with a five-point Likert agreement scale. Examples are: “collective activities are important to keep the beach litter-free” and “everyone is responsible for removing litter from the beach, including me.” An additional item was also included: “only those who originally pollute the beach are responsible for removing litter from the beach.” As stated in Lucrezi and Digun-Aweto (2020), perceiving others such as the local government to be responsible for removing litter can be negatively linked to participation in beach litter removal campaigns. In contrast, perceived personal responsibility can encourage such participation and also reflects the notion of perceived behavioral control, i.e., “it is up to me whether I do this rather than other people or contextual factors.” This is regarded as one of the best direct predictors of behavior change (Klöckner, 2013; Ashley et al., 2019). Furthermore, pro-environmental attitudes in general were measured with the revised version of the ten-item New Ecological Paradigm scale (NEP; Dunlap et al., 2000), that is adapted for children and adolescents (Manoli et al., 2007), with a five-point Likert agreement scale. We used the multidimensional purpose of the NEP scale to evaluate three factors, i.e., the extent to which one endorses the rights of nature (Rights of Nature; e.g. “plants and animals have as much right as people to live”), recognizes the possibility of an eco-crisis (Eco-Crisis; e.g. “if things do not change, we will have a big disaster in the environment soon”), and rejects human exemptionalism (Human Exemptionalism; e.g. “people are clever enough to keep from ruining the earth”; reverse-coded). In our sample, all three factors present low reliability scores for the pre-survey (Cronbach’s alpha = 0.24 for Rights of Nature, 0.49 for Eco-Crisis, and 0.33 for Human Exemptionalism) and for the post-survey (Cronbach’s alpha = 0.13 for Rights of Nature, 0.60 for Eco-Crisis, and 0.37 for Human Exemptionalism).

Behaviors towards marine litter were measured with items displaying litter-reducing behaviors, used in Hartley et al. (2015) and Locritani et al. (2019). Participants rated how often in the past week they “disposed of litter properly,” “picked up litter on the beach,” “bought goods with less packaging,” “encouraged family and friends to do any or all of the things above,” and “avoided using plastic bags in the supermarket” (added item), using a Likert scale from 1 (*never*) to 5 (*a great deal*). To evaluate pro-environmental behavioral intentions, participants had to rate how often in the future they will engage in participation in beach clean-ups (based on Wyles et al., 2017), and other more generic pro-environmental behaviors, namely buying products with less packaging, recycling, and re-using plastic bags. Response options were *never*, *rarely*, *occasionally*, *a moderate amount*, and *a great deal*.

#### 2.2.3. Well-being and nature connectedness

Participants’ well-being was assessed with the Short Warwick Edinburgh Mental Well-Being Scale (SWEMWBS; Stewart-Brown et al., 2009), which is a seven-item scale that measures both eudaimonic and hedonic well-being. Eudaimonic well-being refers to living in accordance with one’s true self (Waterman, 1993) and is typically conceptualized with six dimensions, namely autonomy, environmental mastery, personal growth, positive relationships, purpose in life, and self-acceptance (Ryff and Keyes, 1995). Hedonic well-being is defined as a state of positive affect and absence of negative affect (Kahneman et al., 1999), and is often measured by life satisfaction and happiness. The SWEMWBS was developed by the Universities of Warwick, Edinburgh, and Leeds in conjunction with NHS Health Scotland. Participants had to rate the frequency of certain thoughts and feelings experienced in the past week, with response options going from 1 (*never*) to 5 (*always*). The SWEMWBS is shown to have acceptable reliability in our sample, as Cronbach’s alpha was 0.73 for the pre-survey and 0.80 for the post-survey. To measure specifically hedonic well-being, participants were also asked to rate their happiness with a ten-point scale going from *extremely unhappy* to *extremely happy*. Nature connectedness was assessed with the use of the Nature Connection Index (NCI; Richardson et al., 2019). The NCI is a six-item scale that measures a person’s sense of connection with nature, using items such as “being in nature makes me very happy” or “I feel part of nature.” Participants responded with a seven-point Likert agreement scale. The NCI is suitable for both children and adults and is found to have good reliability in our sample. Cronbach’s alpha was 0.86 for the pre-survey and 0.89 for the post-survey.

In the post-survey, participants were also asked to indicate how satisfied they were with the general COLLECT project with a ten-point scale going from *very unsatisfied* to *very satisfied,* such as in Wyles et al. (2017). The meaningfulness of the COLLECT project was measured with participants rating to what extent the project was worthwhile and meaningful to them, using a scale from 1 (*not at all*) to 5 (*extremely*; based on Wyles et al., 2017). Finally, participants also had to select three adjectives that they thought best described the COLLECT project, from the following list: inspiring, boring, informative, pointless, enjoyable, tiring, motivating, frustrating, gratifying, and challenging.

### 2.3. Procedure

School recruitment was largely based on ongoing collaborations between the member institutes of POGO and science teachers. The headteacher of each school received an official invitation letter from POGO, outlining the project and the activities that would take place. The science teacher selected which classes of students would participate, with no prior knowledge of the content of the surveys and their aim. Parents of the participating students then received an information letter (Catarino et al., 2023). The headteacher and the parents of all underage students were requested to give their informed consent for the student to participate in COLLECT and to take part in the surveys. We also required their consent to take photos or video recordings of the students and to use them for educational and outreach purposes. In addition, all students that were 16 years old or older were required to give their personal informed consent to participate in the surveys.

The pre-survey was handed out to the students during the information session, before the presentation of the project, and typically took about 15 min to complete. The survey contained first a short introduction, informing the students that the aim of the survey was to understand the different impacts of the project. Students were then asked to compose a unique participant code that would enable to link their answers from the pre- and post-surveys, without direct identification of the student. The code consisted of their birth month, followed by the first two letters of their first name and surname. The post-survey was completed approximatively 1 month later, allowing at least 1 week to pass after the classroom processing of the sampled plastic. It is important to note that in Cabo Verde, Côte d’Ivoire and Morocco, the post-surveys were completed at the end of the second season (i.e., spring), 5/6 months after completion of the pre-surveys. A debriefing of the project’s results will be provided to the students and teachers in the format of flyers or visually attractive posters. The collection and analysis of the surveys has been approved by the ethical committee of the Faculty of Psychology and Educational Sciences of Ghent University (ref: 2021/65).

### 2.4. Statistical analysis

Due to the use of ordinal data, we evaluated the effects of the COLLECT project on the participants with Wilcoxon’s matched-pairs signed ranks test, which is a non-parametric statistical method that can determine whether there is a median difference between paired observations. If the assumption that the distribution of differences is symmetrically shaped was violated, then a sign test was employed instead. To evaluate differences between perceived impacts, perceived causes, self-reported litter-reducing behaviors, and attitudes towards beach removal at baseline, we used a Friedman test with pairwise comparisons. We analyzed the intervention effects for each country separately due to unequal group sizes and heterogeneity of covariances. Results from the Moroccan schools could not be included in the analyses because only one participant completed both pre- and post-surveys, due to the disruptions imposed by COVID-19 restrictions in place. For each significant effect we examined whether there was an influence of age, gender, or visit frequency to the beach. We used Spearman correlations to evaluate the relationships between age, coastal visit frequency, and the shift in variables from pre- to post-surveys. For any effects of gender, we used Mann–Whitney *U*-tests to compare the shifts in variables from pre- to post-surveys between men and women. The effects of gender on results from Cabo Verde and Côte d’Ivoire could not be assessed due to a low percentage of men who completed both pre- and post-surveys (6 and 18% respectively).

## 3. Results

### 3.1. Awareness and knowledge of marine litter

#### 3.1.1. Problem awareness and concern

**Figure 1:**
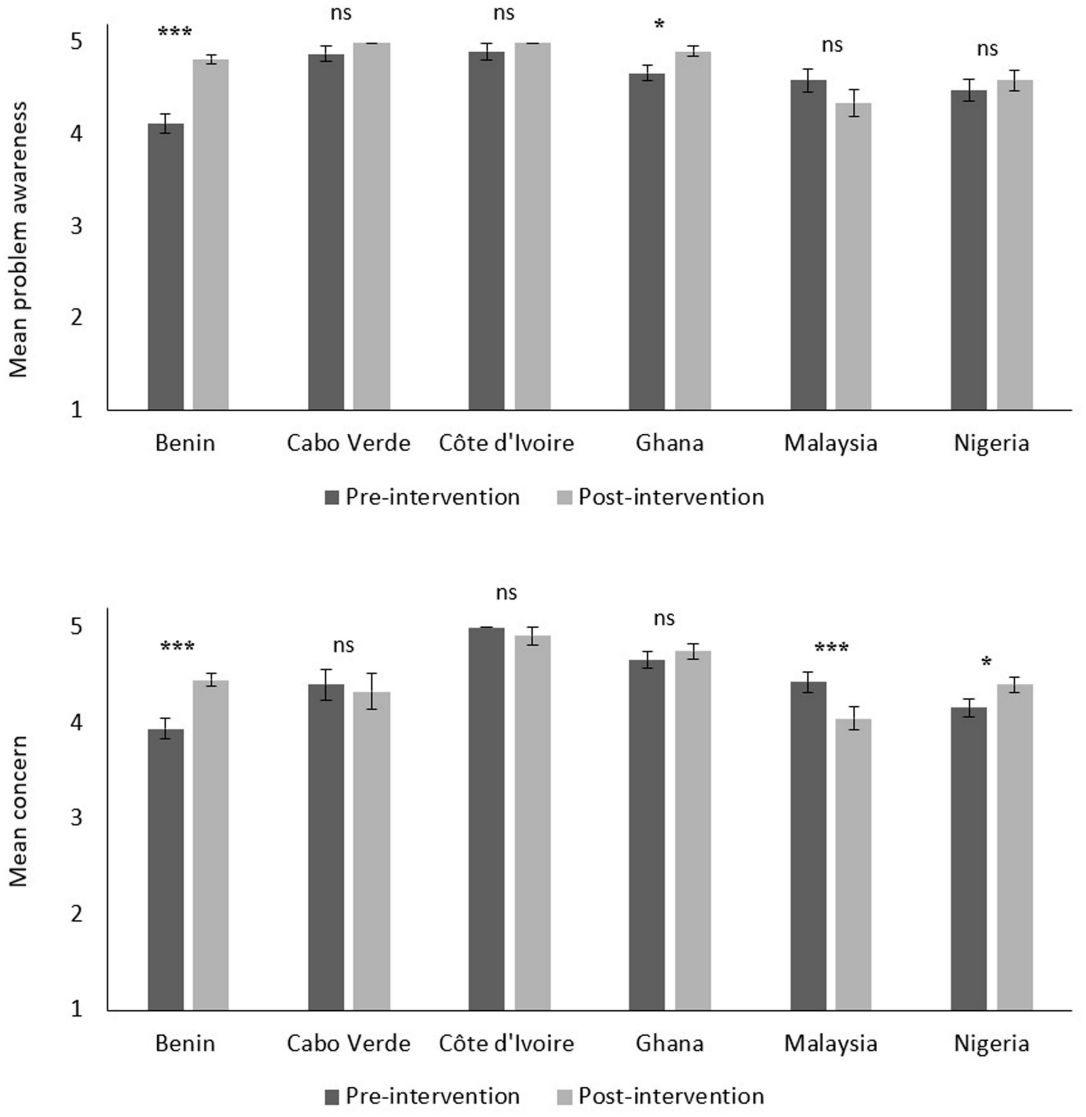
Student’s perceptions of marine litter as a problem (top graph) and concern towards marine litter (bottom graph) per country, at pre- and post-intervention (1–5 scale: *strongly disagree*–*strongly agree*). Error bars represent standard error. ^*^p < 0.05; ^***^p < 0.001; ns = not significant.

Results demonstrate a high perception of marine litter as a problem at baseline in all participating countries (*M* = 4.53). This perception significantly increased at post-intervention in Benin (+14%; *p* < 0.001) and Ghana (+4.8%; *p* = 0.021; Figure 1). Students also considered marine litter to be more problematic in their own country than in other neighboring countries (*M* = 3.42), with a significant increase at post-intervention in Benin (+9.2%; *p* = 0.013). Nonetheless, students perceived marine litter as a future environmental threat rather than a present one (*M* = 3.69) and this did not significantly change at post-intervention for any of the countries. In terms of concern towards marine litter, a high baseline was reported overall (*M* = 4.34). At post-intervention, concern significantly increased in Nigeria (+4.8%; *p* = 0.011) and Benin (+10.2%; *p* < 0.001). In contrast, students in Malaysia reported a significantly lower concern at post-intervention (−7.6%; *p* < 0.001; Figure 1).

#### 3.1.2. Perceived impacts and causes

Baseline responses indicated that students perceived marine litter as adverse for marine wildlife (*M* = 4.57), tourism (*M* = 4.14), human health (*M* = 4.49), fishing activities (*M* = 4.22), and the appearance of the coast (*M* = 4.29). As Friedman tests demonstrated that these impacts were perceived differently at baseline for each country, Bonferroni-corrected pairwise comparisons were performed to evaluate these differences. Marine litter was perceived as having greater negative impacts on marine wildlife and human health than on the appearance of the coast, tourism, and fishing activities in Nigeria, Benin, and Cabo Verde. No significant differences were found between the perceived impacts in Côte d’Ivoire and Ghana. In Malaysia, negative impacts of marine litter were perceived to be greater on marine wildlife in comparison to tourism, human health, and fishing activities. A more detailed analysis of each country is available in Supplementary Materials. Furthermore, perceptions of the negative impacts on marine wildlife increased significantly at post-intervention in Benin (*p* < 0.001). Perception of the negative impacts on tourism also significantly increased after the intervention, in Nigeria (*p* = 0.041), Benin (*p* < 0.001), and Cabo Verde (*p* = 0.004). Additionally, a significant increase was found in perceived negative impacts on fishing activities in Benin (*p* < 0.001), and on the appearance of the coast in Nigeria (*p* = 0.041) and Benin (*p* < 0.001). In contrast, a significant decrease in perceived negative impacts on fishing activities was found in Malaysia at post-intervention (*p* = 0.045). This shift in perception was found to significantly differ according to gender (*p* = 0.01), with males reporting a decreased perception (−13.6%) and females reporting a slightly increased perception (+2.2%).

In regard to perceived causes, students perceived, at baseline, that marine litter is due to people dropping litter (*M* = 4.37), not enough bins (*M* = 3.32), businesses and fishing activities (*M* = 3.54), too much packaging (*M* = 3.54), and rivers discharging wastes into the sea (*M* = 3.34). For every country, Friedman tests indicate that these causes were perceived differently at baseline, thus we conducted Bonferroni-corrected pairwise comparisons to assess these differences. A common difference found in all countries was that people dropping litter was perceived as a greater cause of marine litter than all other causes. In Malaysia and Nigeria, product packaging and businesses and fishing activities were perceived to contribute more to marine litter than the lack of bins, whereas in Benin, product packaging and lack of bins were perceived as greater causes than businesses and fishing activities. Additionally, a lack of bins was perceived as a greater cause than rivers discharging wastes into the sea, in Ghana and Benin. A more detailed analysis of each participating country can be found in the Supplementary Materials. Post-intervention responses indicate significant increases for every perceived cause of marine litter, with the exception of lack of bins for which there were no significant changes. The perception of people dropping litter as a cause of marine litter significantly increased in Benin (*p* = 0.017) and Nigeria (*p* = 0.031) but decreased in Malaysia (*p* = 0.003). The perception of the role of businesses and fishing activities also significantly increased in Benin (*p* = 0.008), Cabo Verde (*p* = 0.005), and Ghana (*p* = 0.026). Students’ perceptions about the extent that too much product packaging contribute to marine litter significantly increased in Benin (*p* = 0.014), Cabo Verde (*p* = 0.019), Côte d’Ivoire (*p* = 0.032), and Ghana (*p* = 0.018). Finally, the perception of rivers discharging wastes into the sea as a cause of marine litter significantly increased in Côte d’Ivoire (*p* = 0.035) and Ghana (*p* = 0.004). Visit frequency to the beach was found to negatively correlate with the difference between pre and post-intervention perceptions of rivers discharging wastes into the sea in Ghana (*r_s_* = −0.387, *p* = 0.029), meaning that those who visit the beach more frequently tended to have decreased perceptions after taking part in COLLECT.

#### 3.1.3. Knowledge of marine litter

A higher percentage of students in Ghana (+23%) and Côte d’Ivoire (+12%) stated the correct response to the degradation time of a plastic bottle after taking part in COLLECT. This was not the case in other countries, as there was an increase in the percentage of students responding “more than 100 years” instead. In terms of perceived plastic salience, results indicate that students had a high perception of plastic salience at baseline. The majority (70%) of students considered plastic to represent more than 50% of marine litter at baseline. This perception significantly increased at post-intervention in Benin (*p* = 0.002). About 83% of the students responded that plastics constituted the most common type of litter found on their local coastline and the percentage remained similar at post-intervention (85%). About 64% of the students believed that plastic bottles had increased between 35 and 75% over the last 10 years. No significant changes concerning that assumption were found in either of the countries at post-intervention. Finally, about 33% of the students responded that on average there were between two to five pieces of litter per meter found on their local coastline, and 48.5% responded there were five pieces or more. Students in Benin perceived a significantly greater number of litter pieces per meter at post-intervention (*p* = 0.014).

### 3.2. Attitudes and behaviors towards marine litter and the environment

#### 3.2.1. Attitudes towards beach litter removal

At baseline, students reported low agreement that only those who originally pollute the beach are responsible for removing beach litter (*M* = 2.42), moderate agreement that it is the responsibility of the local government (*M* = 3.25) and the local community (*M* = 3.73) for removing beach litter and high agreement that everyone is responsible for beach litter removal, including themselves (i.e., collective responsibility) (*M* = 4.39). They also reported high agreement that collective activities are important to keep the beach litter-free (*M* = 4.29). Friedman tests demonstrate significant differences between these items, at baseline, in every country. We therefore performed Bonferroni-corrected pairwise comparisons to evaluate these differences. In all countries, beach litter removal was more perceived to be everyone’s responsibility, rather than the responsibility of only the original polluters. Collective responsibility was also more perceived than the responsibility of the local government, in all countries except for Benin. In Nigeria and Ghana, collective responsibility was more perceived than the responsibility of the local community as well. A more detailed analysis of each country can be found in the Supplementary Materials. Furthermore, post-intervention responses in Benin indicate a significantly increased perception that the local community is responsible for beach litter removal (*p* = 0.007) whereas responses in Malaysia indicate a significantly decreased perception (*p* = 0.032). This contrast is also shown in regards to the perception that collective activities are important to keep the beach litter-free, i.e., increased perception in Benin (*p* < 0.001) and decreased perception in Malaysia (*p* = 0.029). Finally, students in Benin also reported a significantly increased perception of collective responsibility towards beach litter removal at post-intervention (*p* < 0.001).

#### 3.2.2. Pro-environmental attitudes

With regard to pro-environmental attitudes, students reported a high endorsement of the rights of nature (*M* = 3.91), a high recognition of the possibility of an eco-crisis (*M* = 4.08), and a low rejection of human exemptionalism (*M* = 2.73), at baseline. No significant changes at post-intervention were found in the endorsement of the rights of nature in any of the countries. A significant increase in the recognition of the possibility of an eco-crisis was found in Benin (+5.8%; *p* = 0.001), accompanied with a contrasting significant decrease in the rejection of human exemptionalism (−4.9%; *p* = 0.045). It is important to keep in mind however that the three factors describing pro-environmental attitudes presented low reliability scores.

#### 3.2.3. Self-reported litter-reducing behaviors

Baseline responses indicate that students reported a moderate frequency of buying goods with less packaging (*M* = 2.81), avoiding the use of plastic bags in the supermarket (*M* = 2.67), and encouraging family and friends to engage in these actions (*M* = 2.78). A low frequency was reported in terms of picking up litter on the beach (*M* = 1.92), and a moderate frequency was reported in terms of disposing litter properly (*M* = 3.54). For every country, a Friedman test demonstrates significant differences between these behaviors at baseline, thus we conducted Bonferroni-corrected pairwise comparisons to assess these differences. Students reported significantly greater levels of appropriate litter disposal than picking up litter on the beach (all countries), buying goods with less packaging (Nigeria and Malaysia), avoiding plastics bags (Nigeria, Cabo Verde, and Ghana), and encouraging family and friends to act (Nigeria, Benin, and Malaysia). Buying goods with less packaging and encouraging family and friends to act were performed more frequently than picking up litter on the beach in Nigeria, Benin, Côte d’Ivoire, and Ghana. Additionally, these actions were performed more frequently than avoiding the use of plastic bags in Nigeria. A more detailed analysis of each country can be found in the Supplementary Materials. After participating in COLLECT, students in Benin reported a significantly higher frequency of appropriate litter disposal (*p* < 0.001), of encouragement of family and friends (*p* < 0.001), and of buying goods with less packaging (*p* < 0.001). This increase in buying goods with less packaging was found to be positively correlated with coastal visit frequency (*r_s_* = 0.284, *p* = 0.022). Furthermore, at post-intervention students in Nigeria (*p* < 0.001), Benin (*p* < 0.001), and Ghana (*p* = 0.005) reported a significant increase in picking up litter on the beach. Finally, a significant increase in avoiding the use of plastic bags in the supermarket was also reported by students in Benin (*p* < 0.001), Cabo Verde (*p* = 0.039), and Ghana (*p* = 0.001). Table 2 illustrates the pre- and post-intervention means of each self-reported litter-reducing behavior, per country.

**Table 2:** Means (and SD) for self-reported litter-reducing behaviors per country, at pre- and post-intervention (1–5 scale: *never*–*a great deal*).

Self-reported litter-reducing behaviors, per country	Pre-intervention M (SD)	Post-intervention M (SD)
Benin		
Disposed of litter properly	2.92 (1.39)	3.74 (1.25)***
Picked up litter on the beach	1.32 (0.78)	2.56 (1.03)***
Bought goods with less packaging	2.28 (1.02)	3.37 (1.19)***
Avoided using plastic bags in the supermarket	2.31 (1.10)	3.34 (1.18)***
Encouraged family and friends to act	1.91 (1.01)	3.46 (1.26)***
Cabo Verde		
Disposed of litter properly	3.93 (1.27)	4.36 (0.84)
Picked up litter on the beach	2.64 (1.28)	3.00 (0.96)
Bought goods with less packaging	2.92 (0.64)	2.92 (0.76)
Avoided using plastic bags in the supermarket	2.50 (1.02)	3.36 (1.08)*
Encouraged family and friends to act	3.14 (1.23)	3.86 (1.10)
Côte d’Ivoire		
Disposed of litter properly	3.36 (1.63)	3.64 (1.36)
Picked up litter on the beach	2.27 (1.42)	3.09 (1.14)
Bought goods with less packaging	3.09 (1.22)	3.55 (1.04)
Avoided using plastic bags in the supermarket	2.91 (1.22)	3.55 (1.51)
Encouraged family and friends to act	3.70 (1.49)	3.90 (1.29)
Ghana		
Disposed of litter properly	3.91 (1.23)	4.19 (1.03)
Picked up litter on the beach	2.48 (1.02)	3.38 (1.15)**
Bought goods with less packaging	3.43 (1.01)	3.70 (1.12)
Avoided using plastic bags in the supermarket	2.81 (1.51)	3.52 (1.36)**
Encouraged family and friends to act	3.48 (1.21)	3.87 (1.10)
Malaysia		
Disposed of litter properly	3.92 (0.98)	3.89 (0.87)
Picked up litter on the beach	2.24 (1.16)	2.35 (0.92)
Bought goods with less packaging	2.84 (1.09)	3.05 (0.99)
Avoided using plastic bags in the supermarket	3.78 (1.29)	3.65 (1.18)
Encouraged family and friends to act	2.62 (1.16)	2.54 (1.17)
Nigeria		
Disposed of litter properly	3.85 (0.96)	3.87 (1.15)
Picked up litter on the beach	1.49 (0.85)	2.30 (1.13)***
Bought goods with less packaging	2.73 (1.06)	2.95 (1.24)
Avoided using plastic bags in the supermarket	1.97 (1.13)	2.03 (1.10)
Encouraged family and friends to act	2.69 (1.30)	2.84 (1.30)

^*^p < 0.05; ^**^p < 0.01; ^***^p < 0.001.

Post-intervention means in bold are signficiantly higher compared to pre-intervention means.

#### 3.2.4. Pro-environmental behavioral intentions

**Figure 2:**
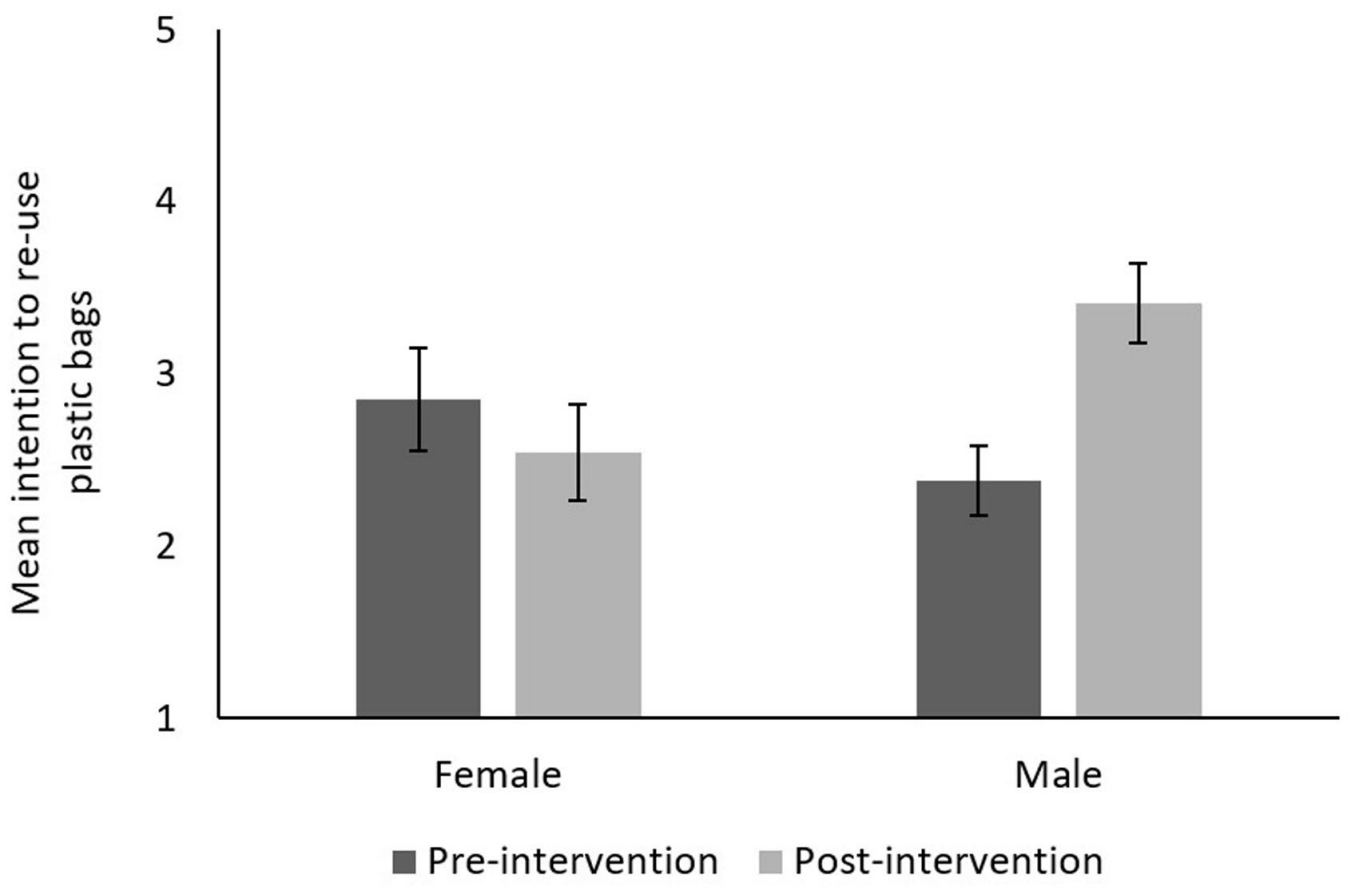
Mean behavioral intention to re-use plastic bags in Benin, per gender, at pre- and post-intervention (1–5 scale: *never*–*a great deal*). Error bars represent standard error.

At baseline, students reported moderate intention to participate in future beach clean-ups (*M* = 3.40), to buy products with less packaging (*M* = 3.53), to recycle (*M* = 3.82), and to re-use plastic bags (*M* = 3.57). After participating in COLLECT, the intention to participate in future beach clean-ups significantly increased for students in Benin (+13.4; *p* = 0.001) and Ghana (+9.6%; *p* = 0.019). Nonetheless, the change in intention to participate in beach clean-ups was found to be negatively correlated with age in Benin (*r_s_* = −0.248, *p* = 0.046) and to be positively correlated with age in Ghana (*r_s_* = 0.357, *p* = 0.049). Older students in Benin therefore expressed less willingness to participate in beach clean-ups at post-intervention, whereas older students in Ghana expressed more willingness. Furthermore, students in Benin reported a significant increase in the intention to buy products with less packaging (+9.2%; *p* = 0.003), to recycle (+12.8%; *p* = 0.002), and to re-use plastic bags (+9.8%; *p* = 0.008). However, the shift in intention to re-use plastic bags was found to significantly differ according to gender (*p* < 0.001), with males reporting an increased intention (+20.6%) and females reporting a slightly decreased intention (−6.2%), at post-intervention (Figure 2).

### 3.3. Well-being and nature connectedness

**Figure 3:**
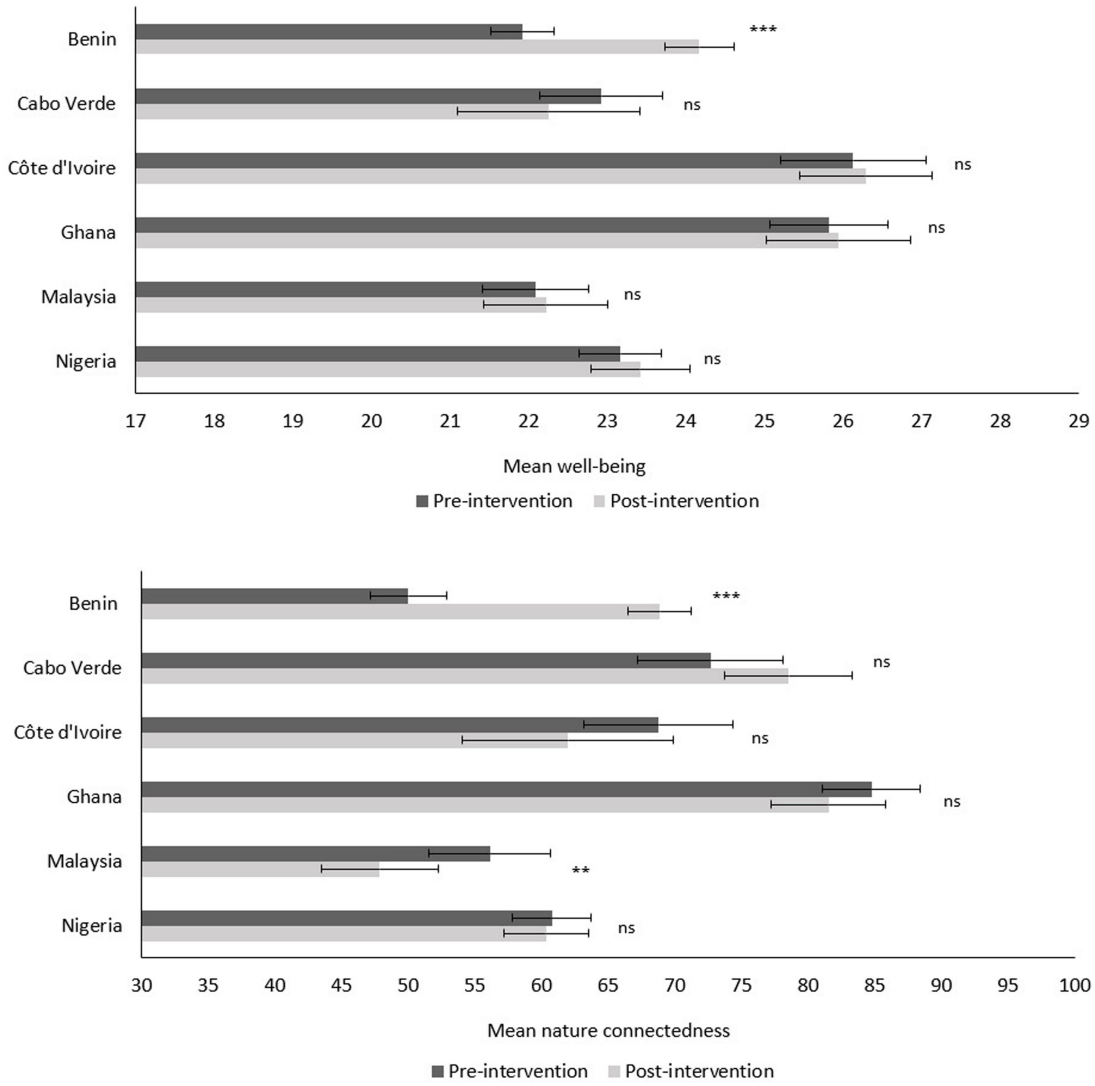
Student’s well-being (top graph; min = 7, max = 35) and nature connectedness (bottom graph; min = 0, max = 100) per country, at pre- and post-intervention. Error bars represent standard error. ^**^p < 0.01; ^***^p < 0.001; ns = not significant.

Differences between pre- and post-intervention mean scores of well-being were evaluated with a paired samples t-test for each country. Only students in Benin reported a significant increase in well-being after participating in COLLECT (+6.4%; *p* < 0.001; Figure 3). Change in happiness was assessed with a Wilcoxon’s matched-pairs signed ranks test for each country. In parallel with well-being, students in Benin demonstrated a significant increase in happiness at post-intervention (+12.3%; *p* < 0.001). Furthermore, at baseline, students displayed an average of 60.78 (*SD* = 25.8) on an index going from 0 to 100 for nature connectedness. Differences between pre- and post-intervention mean scores of nature connectedness were evaluated with a paired samples t-test for each country. A significant increase in nature connectedness was found for students in Benin at post-intervention (+18.9%; *p* < 0.001), whereas a significant decrease was found for students in Malaysia (−8.3%; *p* = 0.007; Figure 3).

### 3.4. Satisfaction with COLLECT

Students reported high satisfaction with the COLLECT project, with average scores above nine (out of ten), with the exception of students in Nigeria (*M* = 7.86) and in Malaysia (*M* = 6.65). Students from all countries considered the COLLECT project to be highly worthwhile and meaningful to them (average scores above four, out of five), with the exception of Malaysia (*M* = 3.35). More than 50% of the adjectives used to describe the COLLECT project referred the project to be “motivating,” “informative,” “inspiring,” and “enjoyable.” About 15% referred the project to be “challenging,” “gratifying,” and “tiring.” Finally, 6% of the adjectives attributed the project as “boring” and “frustrating,” and 2% as “pointless.”

Students and/or teachers also had the option to fill in a feedback form evaluating their thoughts and opinions regarding COLLECT. When asked how we could improve the project, teachers from Morocco and Nigeria responded with a common opinion, namely that the project should be expanded in some way (e.g., by increasing the number of participants or schools involved, or replicating the project several times). One teacher in particular expressed a need for “more education, especially among the younger generation, on recycling and renewable energy.” Teachers also reported several aspects of the project that they liked or appreciated, namely the “hands-on” experience of the field activities and the efficiency and coordination of the project. When asked to what extent the project influenced interest in science and a pursuit in a scientific career, about half of the students from Cabo Verde replied positively, e.g., “I’ve always had this curiosity to work in the scientific field and this project gave me the inspiration I needed to really want to continue with this career.” The learning outcomes of the project reported by the students from Cabo Verde included: how to differentiate the types of plastic, how to sample and analyze plastic, the presence of microplastics, how to work as a team, the impacts of plastic on nature and humans, and the importance of reducing plastic consumption. The project was a learning experience for the teachers as well, as teachers stated learning about the presence of plastics that are “too small to see with the naked eye,” the methodology for plastic sampling, and the negative effects of plastic waste on the ecosystem and humans.

## 4. Discussion

The results of the present study demonstrate that the citizen science intervention in COLLECT positively impacted ocean literacy in Benin, Ghana, Nigeria, Cabo Verde, and Côte d’Ivoire, to differing extents. Students in Benin and Ghana also displayed higher pro-environmental intentions after participating in COLLECT. An increased well-being and nature connectedness were found for students in Benin as well. Ocean literacy was conceptualized by awareness and knowledge towards marine litter, self-reported litter-reducing behaviors, and attitudes towards litter removal. In terms of awareness and knowledge, results indicate a high baseline of problem awareness and concern towards marine litter. This is a finding that has been frequently shown in previous studies investigating citizen science projects related to plastic pollution, namely in the United Kingdom (Hartley et al., 2015), in Italy (Locritani et al., 2019), in Chile (Wichmann et al., 2022), and in Denmark (Oturai et al., 2022). High concern towards marine plastic pollution is also prevalent in the general public in Europe, as displayed in a pan-European citizen survey (H2020 SOPHIE Consortium, 2020). The high baseline problem awareness could be linked with the age group of teenagers recruited in our study, as discussed in Locritani et al. (2019). However, younger children were also found to have high problem awareness prior to any intervention (Oturai et al., 2022; Wichmann et al., 2022). Furthermore, students demonstrated a high perceived salience of plastic on the coast prior to participating in COLLECT. More specifically, students already considered plastic to be the most common type of marine litter and perceived a high increase of the proportion of plastic bottles over recent years. Although students perceived marine litter as a problem, they somewhat perceived this problem to be a future environmental threat, rather than a present one, and this did not significantly change after the intervention. This could indicate a form of temporal distancing from the problem (Hartley et al., 2018; Keller et al., 2022), associated with a perceived lack of urgency (Moser and Dilling, 2007). Future interventions should focus on making the urgency of the problem more apparent for the participants.

Students were highly aware of most causes and consequences of marine litter before participating in COLLECT. Similar to Hartley et al. (2015) and Locritani et al. (2019), the negative impact of marine litter on marine wildlife was generally perceived to be greater than the impacts on the appearance of the coast, tourism, and fishing activities. As stipulated by Hartley et al. (2018), this could be due to either the prominent portrayal of marine wildlife being affected by plastic in the media, or the greater value placed on marine wildlife in comparison to other impacts. The latter is consistent with the European public’s strong concern towards the loss of marine biodiversity (H2020 SOPHIE Consortium, 2020). Unlike in Hartley et al. (2015) and Locritani et al. (2019), human health was also regarded as an important negative impact of marine litter. This is perhaps due to the increasing media representation of the health risks of plastics, particularly microplastics (Völker et al., 2020; Catarino et al., 2021). Students’ perceptions of the causes of marine litter were in line with previous findings, i.e., the perception that people dropping litter is the main cause of marine litter (Campbell et al., 2014; Hartley et al., 2015; Van Dyck et al., 2016). After taking part in COLLECT, students demonstrated a better understanding of other causes of marine litter, i.e., the role of businesses and the fishing industry, product packaging, and rivers discharging wastes into the sea. Enhancing public knowledge of the sources of marine litter is essential as these are often disregarded in the public discourse (Pahl et al., 2017). Importantly, despite the high baseline, participating in COLLECT did lead to increased awareness and knowledge of marine litter, in the majority of the countries.

Consistent with Hartley et al. (2015) and Locritani et al. (2019), students reported greater levels of appropriate litter disposal than all other litter-reducing behaviors at baseline. Picking up litter on the beach was reported with the lowest frequency level, however this could be partially due to the low visit frequency to the beach reported by students (with exception of students from Cabo Verde and Côte d’Ivoire). Participating in COLLECT led to an increase in litter-reducing behaviors in four out of the six evaluated countries. This further supports the notion that citizen science projects have the capacity to impact positive behavior change, as well as awareness. Nonetheless, our study measured behavior through self-report items, and it would be beneficial for future research to assess behavior with experimental measures such as behavioral paradigms (Lange, 2022), to boost empirical evidence of an effect on behavior. Furthermore, in regards to attitudes towards beach litter removal, students already displayed a high perceived personal responsibility towards removing litter from the beach prior to participating in COLLECT. Similar to Lucrezi and Digun-Aweto (2020), students considered beach litter removal as more of a shared responsibility rather than a government responsibility. This could indicate a high baseline of perceived behavioral control regarding removal of beach litter, and thereby an initial willingness to participate in local actions such as beach clean-ups. After the COLLECT intervention, students’ perceived responsibilities did not significantly change, with exception to those in Benin and Malaysia.

Beyond having a positive impact on ocean literacy, participating in COLLECT also led to an increase in intentions to engage in more general pro-environmental behaviors, similar to Wyles et al. (2017). More specifically, students in Benin reported increased intentions to buy products with less packaging, to recycle, and to re-use plastic bags. An increased intention to participate in future beach clean-ups was also found for students in Ghana and Benin. These findings indicate that citizen science projects could lead to spillover effects in which impacting behaviors related to litter reduction encourages intentions to act more sustainably in one’s daily life. Moreover, in terms of attitudes, a possible spillover effect could have occurred for students in Benin, in which a positive change in attitudes regarding litter removal (i.e., higher perceived collective responsibility) encouraged a positive increase in pro-environmental attitudes (i.e., higher recognition of the possibility of an eco-crisis).

With exception to students in Benin, participating in COLLECT did not significantly affect pro-environmental attitudes. At baseline, students did report a somewhat high endorsement of rights of nature and a high recognition of a possibility of an eco-crisis. However, this was accompanied with a low rejection of human exemptionalism. To interpret these findings, it is important to consider the cultural context of the participating countries. To a certain extent, the pro-environmental attitudes reflected here resemble those reported by Nigerian students (Ogunbode, 2013), Zimbabwean children (Van Petegem and Blieck, 2006), and Senegalese children (Grúňová et al., 2019). These studies found that participants have both an ecological and utilitarian view of the environment. More specifically, although participants are concerned with the negative effects that humans have on nature, they also show faith in the recovery of nature from human interference and in the possibility of gaining control of nature. Ogunbode (2013) argues that this control of nature is viewed as possible through spiritual empowerment, with the means of religious ritual and negotiation. The low internal consistency of the NEP factors in our sample further demonstrates this dualistic perspective. For example, students show agreement that people should obey the laws of nature but are also supposed to rule over nature, similar to Grúňová et al. (2019). Although the NEP has been rated as the most comprehensive and widely used scale to measure pro-environmental attitudes (Somerwill and Wehn, 2022), our results are consistent with the notion that the perspective utilized by the NEP only partially represents the ecological worldviews within an African context (Ogunbode, 2013). Moreover, possessing anthropocentric values should not be regarded as a barrier towards implementing pro-environmental behaviors. Indeed, recent findings challenge this stereotype by demonstrating a strong expression of pro-environmental behavior in people with anthropocentric values and a high connection with nature (Sockhill et al., 2022). Better understanding of these particular ecological worldviews is thus needed to tailor the interventions and educational modules targeting students from African countries.

Participating in COLLECT did not significantly impact well-being, nor nature connectedness (with exception to students in Benin and Malaysia). The participating countries presented an average baseline in well-being, as their mean scores are comparable to the mean score of the adult population in the United Kingdom (Ng Fat et al., 2017; Figure 3). Nevertheless, we cannot exclude the possibility that there was a positive impact on happiness or nature connectedness immediately after the citizen science activities, as post-intervention measurements were taken 1 week after the activities ended. Additionally, results indicate that the COLLECT project was perceived to be highly worthwhile and meaningful, consistent with findings from Wyles et al. (2017). Perhaps the focus on plastic pollution and the exposure to a littered environment curtailed the potential restorative qualities of the coast and induced negative emotions, including a more pessimistic outlook regarding the future of the environment (Severin et al., 2023). As such, the meaningfulness of the citizen science activities was perhaps not sufficient to counteract this (Wyles et al., 2017). Another possibility could be that students did not perceive the coast as a place of leisure and restoration and therefore did not expect to feel happier or more relaxed after visiting the beach. Indeed, the cultural context most likely also plays a role here in shaping the relationship and interactions with the coast (Wheaton et al., 2020). For example, it is possible that a portion of the students consider the coast as a dangerous place or merely view it as a source of food and resources (Wheaton et al., 2020; Phoenix et al., 2021). Despite their close proximity to the coast, the average visit frequency to the beach was less than once a week. Finally, students participated in COLLECT as part of a school activity, which induces the possibility of perceiving the project as more of an obligation and not as a leisure activity (similar to Wyles et al., 2017). With regards to nature connectedness, the baseline mean score of the students is comparable to the mean score found for children aged 7 to 15 years in the United Kingdom (Richardson et al., 2019). Half of the participating countries displayed even higher mean scores (see Figure 3), indicating a higher than average baseline of nature connectedness. The non-significant impact of COLLECT on nature connectedness is in contrast to findings from Koss and Kingsley (2010), although their study is based on volunteers and not schoolchildren, which therefore increased the likelihood of prior motivation and interest to engage with nature.

### 4.1. Influence of country, age, and gender on intervention effects

Each country presented unique results in terms of the effect of the COLLECT project on ocean literacy, pro-environmental intentions and attitudes, and well-being. Two countries in particular demonstrated contrasting findings, namely Benin and Malaysia. A positive change in almost all outcomes was found for students in Benin, indicating that the COLLECT project was effective in enhancing awareness towards marine litter, motivating students to engage in litter-reducing behaviors and to act more sustainably, and boosting students’ well-being and nature connectedness. It is important to note however that compared to the other countries, students in Benin reported a lower baseline for the majority of the outcomes, and thus possessed greater room for change. In contrast, students in Malaysia reported a negative change in several outcomes, namely in concern towards marine litter, in perceived impacts and causes of marine litter, in attitudes regarding beach litter removal, and in nature connectedness. Students in Malaysia were also the least satisfied with the COLLECT project and reported the lowest rating in terms of its meaningfulness. Importantly, during the field sampling at the beach, students in Malaysia did not collect any macroplastics and could only sample very few microplastics, as the beach was frequently cleaned by the hotels in the area. This means that students did not directly perceive litter at the beach, thereby potentially reducing the perception of the salience of marine litter on the coastline and creating disinterest towards the aims of the project, similar to findings from Oturai et al. (2022). This is in line with the fact that students in Benin collected the most macroplastics compared to other countries (Catarino et al., 2022), and therefore perceived a strong presence of plastics on the coast, which can partly explain the positive outcomes of the project previously mentioned.

Very few differences in intervention effects were found in terms of age and gender. Indeed, Hartley et al. (2018) demonstrate how age and gender are less important predictors of concern towards marine litter than factors such as values and social norms. Apart from influencing the pre- to post-intervention change in intention to participate in beach clean-ups in Benin (negatively) and Ghana (positively), age did not influence the main intervention effects. This could be due to the low age range with the majority of our sample being between 14 and 18 years old. Previous studies reported differences between those aged 7–12 years and those aged 13–16 years (Hartley et al., 2015; Locritani et al., 2019; Oturai et al., 2022), therefore, age effects are more likely to appear when comparing children with adolescents. The effects of participating in COLLECT did not differ according to gender, except for two outcomes. First, the perception of the negative impact of marine litter on fishing activities decreased for males and slightly increased for females, in Malaysia. Malaysian women are typically less involved in fishing activities, especially in the capture of seafood (Siason et al., 2001). We would therefore expect that they would feel less concerned with the negative impact of marine litter however this was the case for men, and not women. Second, male students in Benin reported an increased intention to re-use plastic bags whereas female students reported a slightly decreased intention. This contrast could stem from a gender disparity in which women are typically more responsible for household duties and therefore need stronger motivation to change their habits such as re-using plastic bags to buy groceries.

### 4.2. Limitations and future directions

The present study evaluates the impact of a large-scale citizen science project implemented in multiple countries, presenting a unique opportunity for obtaining diverse knowledge from underrepresented regions. This however also led to several methodological limitations that should be considered. First, although the implementation of the project was standardized as much as possible (Catarino et al., 2023), there are notable differences between the countries. One difference is in regards to the time period between the pre- and post-surveys (i.e., 1 month for Nigeria, Benin, Ghana, and Malaysia; 5/6 months for Cabo Verde, Côte d’Ivoire, and Morocco). Additionally, the surveys were conducted in different languages, leading to potential differences in the interpretation of certain words or expressions between the countries. Nonetheless, results of each country were analyzed separately and thus no statistical comparison between countries was made. Furthermore, as is often the case with follow-up measures, results portray only a subset of the total sample (58%) because not all students completed both the pre- and post-surveys. This led to a reduced sample size and a loss of valuable information. Moreover, the data from the surveys had to be manually transferred to a computer due to the surveys being completed in paper format, and was therefore exposed to potential human error. Finally, although efforts were made to use psychometric scales that were validated in various cultural settings (e.g., the SWEMWBS; Stewart-Brown et al., 2009), it is essential to consider the influence of the cultural context upon the validity of the scales and items included in our study.

For future research conducting citizen science in African regions, we recommend further investigation into how African youth shape their environmental attitudes and perspectives regarding current issues such as plastic pollution. For example, it would be beneficial to first conduct a qualitative study exploring the awareness and attitudes of students and teachers and the perceived barriers hindering behavioral change. The resulting knowledge could then be utilized to integrate educational workshops tailored to these unique perspectives into the global citizen science project. As Wichmann et al. (2022) recommend, these educational modules should inform strategies and competencies for students to adopt to enhance empowerment towards tackling environmental issues. We also recommend to use measurements that have been validated in non-WEIRD populations (Western Educated Industrialized Rich Democratic). Furthermore, to promote empirical evidence of a positive effect of citizen science on attitudes and behavior relating to marine litter, we suggest implementing a longitudinal design to evaluate whether beneficial effects remain in the long-term. Additionally, specifically in terms of beach clean-ups or sampling of beach litter, there should be further research on the influence of the extent of litter present on the beach on subsequent perceptions regarding plastic pollution and motivations for sustainable action (in reference to the negative impacts shown in Malaysia).

### 4.3. Conclusion and implications

In conclusion, the present study highlights the effectiveness of a citizen science intervention to positively impact ocean literacy. Despite a high baseline, students demonstrated greater awareness and a better understanding of the causes and consequences of marine litter, in the majority of the participating countries. Participating in COLLECT also led to an increase in litter-reducing behaviors, such as picking up litter on the beach and avoiding the use of plastic bags. Students reported a high baseline of perceived personal responsibility towards beach litter removal and this did not change at post-intervention. Some shortcomings to the project can be noted. To a certain extent, taking part in COLLECT did not reduce temporal distancing from the issue of marine litter, nor did it positively change pro-environmental attitudes. Moreover, students in Malaysia demonstrated negative shifts in awareness and knowledge, in attitudes towards beach litter removal, and in nature connectedness, possibly due to a lack of perceived salience of marine litter at their local coastline. Nonetheless, participating in COLLECT led to higher pro-environmental behavioral intentions for students in Benin and Ghana, indicating a positive spillover effect. Students from Benin are shown to have benefited the most out of participating in COLLECT as they also demonstrated higher well-being and nature connectedness after the citizen science intervention. The COLLECT project was overall positively perceived by the students and teachers and evaluated as an important learning experience.

In light of these findings, we stress the importance of evaluating the educational and behavioral benefits of citizen science interventions to address plastic pollution on a societal and individual level. Understanding the perceptions regarding marine litter can have important implications for management and policy decision-making. As showcased in Sumeldan et al. (2021), gaining knowledge into how local communities perceive a particular issue can help inform stakeholders on how to best communicate about this issue or on how to adapt strategies targeting behavior change in accordance with the attitudes and motivations of the public. Moreover, displaying empirical evidence of the positive impacts of citizen science on participants enables to enlarge the range of citizen science benefits and encourage researchers and organizations to implement it wherever possible.

## Data availability statement

The datasets presented in this study can be found in online repositories. The names of the repository/repositories and accession number(s) can be found below: Integrated Marine Information System (IMIS), https://doi.org/10.14284/587.

## Ethics statement

The studies involving human participants were reviewed and approved by Ethical Committee of the Faculty of Psychology and Educational Sciences of Ghent University (ref: 2021/65). Written informed consent to participate in this study was provided by the participants’ legal guardian/next of kin.

## Author contributions

MS: conceptualization, methodology, formal analysis, investigation, data curation, writing – original draft, and writing – review and editing. LA: investigation, data curation, resources, project administration, and writing – review and editing. PA: investigation, data curation, and writing – review and editing. FA, MB, AJ-R, MM, IM, PN, OA, ZS, SW, and SZ: investigation, data curation, resources, project administration, and writing – review and editing. FB: resources, project administration, and writing – review and editing. JM: conceptualization, resources, funding acquisition, and writing – review and editing. JN: data curation and writing – review and editing. YS: data curation, resources, and writing – review and editing. AS-H: conceptualization, resources, project administration, funding acquisition, supervision, and writing – review and editing. AB and FR: supervision and writing – review and editing. LK, SS, and GE: conceptualization, methodology, resources, project administration, funding acquisition, supervision, and writing – review and editing. EM: investigation, data curation, resources, project administration, funding acquisition, supervision, and writing – review and editing. AC: conceptualization, methodology, investigation, data curation, resources, project administration, funding acquisition, supervision, writing – review and editing. All authors contributed to the article and approved the submitted version.

## Funding

The COLLECT project has been funded by the Richard Lounsbery Foundation (United States), and by internal funds of the Partnership for the Observation of the Global Ocean (POGO), United Kingdom, and from the Flanders Marine Institute (VLIZ), Belgium. MS is a holder of a PhD fellowship from VLIZ.

## Conflict of interest

The authors declare that the research was conducted in the absence of any commercial or financial relationships that could be construed as a potential conflict of interest.

## Publisher’s note

All claims expressed in this article are solely those of the authors and do not necessarily represent those of their affiliated organizations, or those of the publisher, the editors and the reviewers. Any product that may be evaluated in this article, or claim that may be made by its manufacturer, is not guaranteed or endorsed by the publisher.

## Footnotes

^1^ https://pogo-ocean.org

^2^ litterbase.awi.de

^3^ https://osf.io/vb8tx

^4^ https://osf.io/7e3su/

^5^ www.limesurvey.org

## References

[ref1] Allen N. S. Edge M. Mohammadian M. Jones K. (1994). Physicochemical aspects of the environmental degradation of poly(ethylene terephthalate). Polym. Degrad. Stab. 43, 229–237. doi: 10.1016/0141-3910(94)90074-4

[ref2] Arabi S. Neehaul Y. Sparks C. (2023). “Impacts and threats of marine litter in African seas” in The African Marine Litter Outlook. eds. Maes T. Preston-Whyte F. (Cham: Springer), 91–136.

[ref3] Ashley M. Pahl S. Glegg G. Fletcher S. (2019). A change of mind: applying social and behavioral research methods to the assessment of the effectiveness of ocean literacy initiatives. Front. Mar. Sci. 6:288. doi: 10.3389/fmars.2019.00288

[ref4] Babayemi J. O. Nnorom I. C. Osibanjo O. Weber R. (2019). Ensuring sustainability in plastics use in Africa: consumption, waste generation, and projections. Environ. Sci. Eur. 31:60. doi: 10.1186/s12302-019-0254-5

[ref5] Barnardo T. Ribbink A. (Eds.). (2020). African Marine Litter Monitoring Manual. African Marine Waste Network, Sustainable Seas Trust. Port Elizabeth, South Africa.

[ref6] Bergmann M. Tekman M. Gutow L. (2017). “LITTERBASE: an online portal for marine litter and microplastics and their implications for marine life,” in MICRO. Elsevier: Amsterdam, 106–107.

[ref7] Campbell M. L. Paterson de Heer C. Kinslow A. (2014). Littering dynamics in a coastal industrial setting: the influence of non-resident populations. Mar. Pollut. Bull. 80, 179–185. doi: 10.1016/j.marpolbul.2014.01.015, PMID: 24486045

[ref8] Catarino A. I. Kramm J. Völker C. Henry T. B. Everaert G. (2021). Risk posed by microplastics: scientific evidence and public perception. Curr. Opin. Green Sustain. Chem. 29:100467. doi: 10.1016/j.cogsc.2021.100467

[ref9] Catarino A. I. Mahu E. Severin M. I. Akpetou K. L. Annasawmy P. Asuquo F. E.. (2022). Citizen Observation of Plastic Pollution in Coastal Ecosystems to Address Data Gaps in Marine Litter Distribution [Conference Presentation]. MICRO 2022, Online Atlas Edition: Plastic Pollution from MACRO to Nano, Online.

[ref10] Catarino A. I. Mahu E. Severin M. I. Akpetou L. K. Annasawmy P. Asuquo F. E.. (2023). Addressing data gaps in marine litter distribution: citizen science observation of plastics in coastal ecosystems by high-school students. Front. Mar. Sci. 10:1126895. doi: 10.3389/fmars.2023.1126895

[ref11] Chen H. L. Nath T. K. Chong S. Foo V. Gibbins C. Lechner A. M. (2021). The plastic waste problem in Malaysia: management, recycling and disposal of local and global plastic waste. S. N. Appl. Sci. 3:437. doi: 10.1007/s42452-021-04234-y

[ref12] Connell J. P. Kubisch A. C. (1998). “Applying a theory of change approach to the evaluation of comprehensive community initiatives: Progress, prospects, and problems” in New Approaches to Evaluating Community Initiatives. eds. Connell J. P. Kubisch A. C. Schorr L. B. Weiss C. H. (Washington DC: The Aspen Institute), 15–44.

[ref13] De Rijck K. Schade S. Rubio J.-M. Van Meerloo M. (2020). Best Practices in Citizen Science for Environmental Monitoring. Brussels, European Commission. Luxembourg: European Commission.

[ref14] De Veer D. Drouin A. Fischer J. González C. Holtmann G. Honorato-Zimmer D.. (2022). How do schoolchildren perceive litter? Overlooked in urban but not in natural environments. J. Environ. Psychol. 81:101781. doi: 10.1016/j.jenvp.2022.101781

[ref15] Dunlap R. E. Van Liere K. D. Mertig A. G. Jones R. E. (2000). New trends in measuring environmental attitudes: measuring endorsement of the new ecological paradigm: a revised NEP scale. J. Soc. Issues 56, 425–442. doi: 10.1111/0022-4537.00176

[ref16] Edge M. Hayes M. Mohammadian M. Allen N. S. Jewitt T. S. Brems K.. (1991). Aspects of poly(ethylene terephthalate) degradation for archival life and environmental degradation. Polym. Degrad. Stab. 32, 131–153. doi: 10.1016/0141-3910(91)90047-U

[ref17] Fauziah S. H. Rizman-Idid M. Cheah W. Loh K. H. Sharma S. (2021). Marine debris in Malaysia: a review on the pollution intensity and mitigating measures. Mar. Pollut. Bull. 167:112258. doi: 10.1016/j.marpolbul.2021.112258, PMID: 33839567

[ref18] Geiger S. J. Brick C. Nalborczyk L. Bosshard A. Jostmann N. B. (2021). More green than gray? Toward a sustainable overview of environmental spillover effects: a Bayesian meta-analysis. J. Environ. Psychol. 78:101694. doi: 10.1016/j.jenvp.2021.101694

[ref19] Grúňová M. Sané M. Cincera J. Kroufek R. Hejcmanová P. (2019). Reliability of the new environmental paradigm for analysing the environmental attitudes of Senegalese pupils in the context of conservation education projects. Environ. Educ. Res. 25, 211–221. doi: 10.1080/13504622.2018.1428942

[ref20] H2020 SOPHIE Consortium. (2020). Citizens and the Sea. Public Perceptions of Oceans and Human Health: A 14-Country Pan-European Citizen Survey. H2020 SOPHIE Project. Available at: https://sophie2020.eu/wp/wp-content/uploads/2020/06/Citizens-and-the-Sea-Report_web.pdf (Accessed February 10, 2023).

[ref21] Hartley B. L. Pahl S. Veiga J. Vlachogianni T. Vasconcelos L. Maes T.. (2018). Exploring public views on marine litter in Europe: perceived causes, consequences and pathways to change. Mar. Pollut. Bull. 133, 945–955. doi: 10.1016/j.marpolbul.2018.05.061, PMID: 29910143

[ref22] Hartley B. L. Thompson R. C. Pahl S. (2015). Marine litter education boosts children’s understanding and self-reported actions. Mar. Pollut. Bull. 90, 209–217. doi: 10.1016/j.marpolbul.2014.10.049, PMID: 25467869

[ref23] Hidalgo-Ruz V. Thiel M. (2013). Distribution and abundance of small plastic debris on beaches in the SE Pacific (Chile): a study supported by a citizen science project. Mar. Environ. Res. 87-88, 12–18. doi: 10.1016/j.marenvres.2013.02.015, PMID: 23541391

[ref24] Hooyberg A. Roose H. Grellier J. Elliott L. R. Lonneville B. White M. P.. (2020). General health and residential proximity to the coast in Belgium: results from a cross-sectional health survey. Environ. Res. 184:109225. doi: 10.1016/j.envres.2020.109225 32078817

[ref25] Intergovernmental Oceanographic Commission (2018). Revised Roadmap for the UN Decade of Ocean Science for Sustainable Development (IOC/EC-LI/2 ANNEX 3). Paris: UNESCO.

[ref26] Jambeck J. Geyer R. Wilcox C. Siegler T. R. Perryman M. E. Andrady A. L.. (2015). Plastic waste inputs from land into the ocean. Science 347, 768–771. doi: 10.1126/science.1260352 25678662

[ref27] Jambeck J. Hardesty B. D. Brooks A. L. Friend T. Teleki K. Fabres J.. (2018). Challenges and emerging solutions to the land-based plastic waste issue in Africa. Mar. Policy 96, 256–263. doi: 10.1016/j.marpol.2017.10.041

[ref28] Jorgensen B. Krasny M. Baztan J. (2021). Volunteer beach cleanups: civic environmental stewardship combating global plastic pollution. Sustain. Sci. 16, 153–167. doi: 10.1007/s11625-020-00841-7

[ref29] Kahneman D. Diener E. Schwarz N. (Eds.) (1999). Well-Being: Foundations of Hedonic Psychology. New York: Russell Sage Foundation.

[ref30] Karbalaei S. Golieskardi A. Hamzah H. B. Abdulwahid S. Hanachi P. Walker T. R.. (2019). Abundance and characteristics of microplastics in commercial marine fish from Malaysia. Mar. Pollut. Bull. 148, 5–15. doi: 10.1016/j.marpolbul.2019.07.072 31422303

[ref31] Kawabe L. A. Ghilardi-Lopes N. P. Turra A. Wyles K. J. (2022). Citizen science in marine litter research: a review. Mar. Pollut. Bull. 182:114011. doi: 10.1016/j.marpolbul.2022.114011, PMID: 35964433

[ref32] Keller E. Marsh J. E. Richardson B. H. Ball L. J. (2022). A systematic review of the psychological distance of climate change: towards the development of an evidence-based construct. J. Environ. Psychol. 81:101822. doi: 10.1016/j.jenvp.2022.101822

[ref33] Kelly R. Evans K. Alexander K. Bettiol S. Corney S. Cullen-Knox C.. (2022). Connecting to the oceans: supporting ocean literacy and public engagement. Rev. Fish Biol. Fish. 32, 123–143. doi: 10.1007/s11160-020-09625-9, PMID: 33589856 PMC7875172

[ref34] Klöckner C. A. (2013). A comprehensive model of the psychology of environmental behaviour-a meta-analysis. Glob. Environ. Chang. 23, 1028–1038. doi: 10.1016/j.gloenvcha.2013.05.014

[ref35] Kordella S. Geraga M. Papatheodorou G. Fakiris E. Mitropoulou I. M. (2013). Litter composition and source contribution for 80 beaches in Greece, eastern Mediterranean: a nationwide voluntary clean-up campaign. Aquat. Ecosyst. Health Manage. 16, 111–118. doi: 10.1080/14634988.2012.759503

[ref36] Koss R. S. Kingsley J. Y. (2010). Volunteer health and emotional wellbeing in marine protected areas. Ocean Coast. Manag. 53, 447–453. doi: 10.1016/j.ocecoaman.2010.06.002

[ref37] Krelling A. P. Williams A. T. Turra A. (2017). Differences in perception and reaction of tourist groups to beach marine debris that can influence a loss of tourism revenue in coastal areas. Mar. Policy 85, 87–99. doi: 10.1016/j.marpol.2017.08.021

[ref38] Lange F. (2022). Behavioral paradigms for studying pro-environmental behavior: a systematic review. Behav. Res. Methods 55, 600–622. doi: 10.3758/s13428-022-01825-4, PMID: 35355239

[ref39] Locritani M. Merlino S. Abbate M. (2019). Assessing the citizen science approach as tool to increase awareness on the marine litter problem. Mar. Pollut. Bull. 140, 320–329. doi: 10.1016/j.marpolbul.2019.01.023, PMID: 30803651

[ref40] Lucrezi S. Digun-Aweto O. (2020). “Who wants to join?” visitors’ willingness to participate in beach litter clean-ups in Nigeria. Mar. Pollut. Bull. 155:111167. doi: 10.1016/j.marpolbul.2020.111167, PMID: 32314746

[ref41] Manoli C. C. Johnson B. Dunlap R. E. (2007). Assessing children’s environmental worldviews: modifying and validating the new ecological paradigm scale for use with children. J. Environ. Educ. 38, 3–13. doi: 10.3200/JOEE.38.4.3-13

[ref42] Martin L. White M. P. Hunt A. Richardson M. Pahl S. Burt J. (2020). Nature contact, nature connectedness and associations with health, wellbeing and pro-environmental behaviours. J. Environ. Psychol. 68:101389. doi: 10.1016/j.jenvp.2020.101389

[ref43] Mcllgorm A. Raubenheimer K. Mcllgorm D. E. (2020). “Update of 2009 APEC report on economic costs of marine debris to APEC economies” in Australian National Centre for Ocean Resources and Security (ANCORS) (Australia: University of Wollongong)

[ref44] Miloslavich P. Seeyave S. Muller-Karger F. E. Bax N. J. Ali E. M. Delgado C.. (2019). Challenges for global ocean observation: the need for increased human capacity. J. Oper. Oceanogr. 12, s137–S156. doi: 10.1080/1755876x.2018.1526463

[ref45] Moser S. C. Dilling L. (Eds.) (2007). Creating a Climate for Change: Communicating Climate Change and Facilitating Social Change. Cambridge: Cambridge University Press.

[ref46] Ng Fat L. Scholes S. Boniface S. Mindell J. Stewart-Brown S. (2017). Evaluating and establishing national norms for mental wellbeing using the short Warwick-Edinburgh mental well-being scale (SWEMWBS): findings from the health survey for England. Qual. Life Res. 26, 1129–1144. doi: 10.1007/s11136-016-1454-8, PMID: 27853963 PMC5376387

[ref47] Nubi A. T. Nubi O. A. Franco-Garcia L. Oyediran L. O. (2019). Effective solid waste management: a solution to the menace of marine litter in coastal communities of Lagos state, Nigeria. Afr. J. Environ. Sci. Technol. 13, 104–116. doi: 10.5897/AJEST2018.2588

[ref48] Ocean Conservancy. (2022). The International Coastal Cleanup Report. Available at: https://oceanconservancy.org/wp-content/uploads/2022/09/Annual-Report_FINALWebVersion.pdf (Accessed February 10, 2023).

[ref49] Ogunbode C. A. (2013). The NEP scale: measuring ecological attitudes/worldviews in an African context. Environ. Dev. Sustain. 15, 1477–1494. doi: 10.1007/s10668-013-9446-0

[ref50] Oturai N. G. Pahl S. Syberg K. (2022). How can we test plastic pollution perceptions and behavior? A feasibility study with Danish children participating in the mass experiment. Sci. Total Environ. 806:150914. doi: 10.1016/j.scitotenv.2021.150914 34653473

[ref51] Pahl S. Wyles K. J. (2017). The human dimension: how social and behavioural research methods can help address microplastics in the environment. Anal. Methods 9, 1404–1411. doi: 10.1039/c6ay02647h

[ref52] Pahl S. Wyles K. J. Thompson R. C. (2017). Channelling passion for the ocean towards plastic pollution. Nat. Hum. Behav. 1, 697–699. doi: 10.1038/s41562-017-0204-4, PMID: 31024098

[ref53] Paredes-Coral E. Deprez T. Mokos M. Vanreusel A. Roose H. (2022). The blue survey: validation of an instrument to measure ocean literacy among adults. Mediterr. Mar. Sci. 23, 321–326. doi: 10.12681/mms.26608

[ref54] Phoenix C. Bell S. L. Hollenbeck J. (2021). Segregation and the sea: toward a critical understanding of race and coastal blue space in greater Miami. J. Sport Soc. Issues 45, 115–137. doi: 10.1177/0193723520950536

[ref55] POGO (2021). Taking the Pulse of the Global Ocean: Strategy of the Partnership for the Observation of the Global Ocean. Available at: https://pogo-ocean.org/pogo-strategy (Accessed February 10, 2023).

[ref56] Rambonnet L. Vink S. C. Land-Zandstra A. M. Bosker T. (2019). Making citizen science count: best practices and challenges of citizen science projects on plastics in aquatic environments. Mar. Pollut. Bull. 145, 271–277. doi: 10.1016/j.marpolbul.2019.05.056, PMID: 31590787

[ref57] Rangel-Buitrago N. Williams A. Anfuso G. (2018). Killing the goose with the golden eggs: litter effects on scenic quality of the Caribbean coast of Colombia. Mar. Pollut. Bull. 127, 22–38. doi: 10.1016/j.marpolbul.2017.11.023, PMID: 29475658

[ref58] Rayon-Viña F. Miralles L. Fernandez-Rodríguez S. Dopico E. Garcia-Vazquez E. (2019). Marine litter and public involvement in beach cleaning: disentangling perception and awareness among adults and children, Bay of Biscay, Spain. Mar. Pollut. Bull. 141, 112–118. doi: 10.1016/j.marpolbul.2019.02.034, PMID: 30955715

[ref59] Richardson M. Hunt A. Hinds J. Bragg R. Fido D. Petronzi D.. (2019). A measure of nature connectedness for children and adults: validation, performance, and insights. Sustainability 11:3250. doi: 10.3390/SU11123250

[ref60] Ryan P. G. (2020). The transport and fate of marine plastics in South Africa and adjacent oceans. S. Afr. J. Sci. 116:7677. doi: 10.17159/sajs.2020/7677

[ref61] Ryff C. D. Keyes C. L. M. (1995). The structure of psychological well-being revisited. J. Pers. Soc. Psychol. 69, 719–727. doi: 10.1037/0022-3514.69.4.719 7473027

[ref62] Saghir J. Santoro J. (2018). Urbanization in Sub-Saharan Africa. Meeting Challenges by Bridging Stakeholders. Washington, DC, USA: Center for Strategic & International Studies.

[ref63] Schoedinger S. Cava F. Strang C. Tuddenham P. (2005). Ocean Literacy through Science Standards. Proceedings of OCEANS 2005 MTS/IEEE, 1, 736–740. (Accessed February 10, 2023).

[ref64] Severin M. I. Catarino A. I. Everaert G. Mahu E. (2021a). COLLECT Project [Pre-Registration] Open Science Framework Registries.

[ref65] Severin M. I. Hooyberg A. Everaert G. Catarino A. I. (2023). “Using citizen science to understand plastic pollution: implications for science and participants” in Living in the Plastic Age. Perspectives from Humanities, Social Sciences and Environmental Sciences. eds. Kramm J. Völker C. (Frankfurt, New York: Campus Publisher), 133–168.

[ref66] Severin M. I. Raes F. Notebaert E. Lambrecht L. Everaert G. Buysse A. (2022). A qualitative study on emotions experienced at the coast and their influence on well-being. Front. Psychol. 13:902122. doi: 10.3389/fpsyg.2022.902122, PMID: 35756269 PMC9226434

[ref67] Severin M. I. Vandegehuchte M. B. Hooyberg A. Buysse A. Raes F. Everaert G. (2021b). Influence of the belgian coast on well-being during the COVID-19 pandemic. Psychol. Belgica 61, 284–295. doi: 10.5334/PB.1050, PMID: 34621529 PMC8462480

[ref68] Siason I. Tech E. Matics Choo P. S Shariff M. Heruwati E. S. Susilowati T.. (2001). Women in Fisheries in Asia [Conference Presentation]. Global Symposium on Women in Fisheries: Sixth Asian Fisheries Forum, Kaohsiung, Taiwan.

[ref69] Sockhill N. J. Dean A. J. Oh R. R. Y. Fuller R. A. (2022). Beyond the ecocentric: diverse values and attitudes influence engagement in pro-environmental behaviours. People Nat 4, 1500–1512. doi: 10.1002/pan3.10400

[ref70] Somerwill L. Wehn U. (2022). How to measure the impact of citizen science on environmental attitudes, behaviour and knowledge? A review of state-of-the-art approaches. Environ. Sci. Eur. 34, 1–29. doi: 10.1186/s12302-022-00596-1

[ref71] Stewart-Brown S. Tennant A. Tennant R. Platt S. Parkinson J. Weich S. (2009). Internal construct validity of the Warwick-Edinburgh mental well-being scale (WEMWBS): a Rasch analysis using data from the Scottish health education population survey. Health Qual. Life Outcomes 7:15. doi: 10.1186/1477-7525-7-15, PMID: 19228398 PMC2669062

[ref72] Sumeldan J. D. C. Richter I. Avillanosa A. L. Bacosa H. P. Creencia L. A. Pahl S. (2021). Ask the locals: a community-informed analysis of perceived marine environment quality over time in Palawan, Philippines. Front. Psychol. 12:661810. doi: 10.3389/fpsyg.2021.661810, PMID: 34447327 PMC8382879

[ref73] The Economist. (2019). Ever More Countries are Banning Plastic Bags. De Economist. Available at: https://www.economist.com/graphic-detail/2019/07/24/ever-more-countries-are-banning-plastic-bags (Accessed February 10, 2023).

[ref74] Thiel M. Bravo M. Hinojosa I. A. Luna G. Miranda L. Núñez P.. (2011). Anthropogenic litter in the SE Pacific: an overview of the problem and possible solutions. Rev. Gestão Costeira Integr. 11, 115–134. doi: 10.5894/rgci207

[ref75] Thøgersen J. Ölander F. (2003). Spillover of environment-friendly consumer behaviour. J. Environ. Psychol. 23, 225–236. doi: 10.1016/S0272-4944(03)00018-5

[ref76] Tudor D. T. Williams A. T. (2006). A rationale for beach selection by the public on the coast of Wales. UK. Area 38, 153–164. doi: 10.1111/j.1475-4762.2006.00684.x

[ref77] Van Cauwenberghe L. Janssen C. R. (2014). Microplastics in bivalves cultured for human consumption. Environ. Pollut. 193, 65–70. doi: 10.1016/j.envpol.2014.06.010, PMID: 25005888

[ref78] Van Dyck I. P. Nunoo F. K. Lawson E. T. (2016). An empirical assessment of marine debris, seawater quality and littering in Ghana. J. Geosci. Environ. Protect. 4, 21–36. doi: 10.4236/gep.2016.45003

[ref79] Van Petegem P. Blieck A. (2006). The environmental worldview of children: a cross-cultural perspective. Environ. Educ. Res. 12, 625–635. doi: 10.1080/13504620601053662

[ref80] Völker C. Kramm J. Wagner M. (2020). On the creation of risk: framing of microplastics risks in science and media. Global Chall. 4:1900010. doi: 10.1002/gch2.201900010

[ref81] Waterman A. S. (1993). Two conceptions of happiness: contrasts of personal expressiveness (eudaimonia) and hedonic enjoyment. J. Pers. Soc. Psychol. 64, 678–691. doi: 10.1037/0022-3514.64.4.678

[ref82] Wheaton B. Waiti J. Cosgriff M. Burrows L. (2020). Coastal blue space and wellbeing research: looking beyond western tides. Leis. Stud. 39, 83–95. doi: 10.1080/02614367.2019.1640774

[ref83] White M. P. Elliott L. R. Gascon M. Roberts B. Fleming L. E. (2020). Blue space, health and well-being: a narrative overview and synthesis of potential benefits. Environ. Res. 191:110169. doi: 10.1016/j.envres.2020.110169, PMID: 32971082

[ref84] Wichmann C. S. Fischer D. Geiger S. M. Honorato-Zimmer D. Knickmeier K. Kruse K.. (2022). Promoting pro-environmental behavior through citizen science? A case study with Chilean schoolchildren on marine plastic pollution. Mar. Policy 141:105035. doi: 10.1016/j.marpol.2022.105035

[ref85] Williams A. T. Rangel-Buitrago N. G. Anfuso G. Cervantes O. Botero C. M. (2016). Litter impacts on scenery and tourism on the Colombian North Caribbean coast. Tour. Manag. 55, 209–224. doi: 10.1016/j.tourman.2016.02.008

[ref86] World Health Organization. (2022). Dietary and Inhalation Exposure to Nano- and Microplastic Particles and Potential Implications for Human Health. Available at: https://www.who.int/publications/i/item/9789240054608 (Accessed February 10, 2023).

[ref87] Wyles K. J. Pahl S. Holland M. Thompson R. C. (2017). Can beach cleans do more than clean-up litter? Comparing beach cleans to other coastal activities. Environ. Behav. 49, 509–535. doi: 10.1177/0013916516649412, PMID: 28546642 PMC5431367

[ref88] Wyles K. J. Pahl S. Thomas K. Thompson R. C. (2016). Factors that can undermine the psychological benefits of coastal environments: exploring the effect of tidal state, presence, and type of litter. Environ. Behav. 48, 1095–1126. doi: 10.1177/0013916515592177, PMID: 27807388 PMC5066481

[ref89] Wyles K. J. White M. P. Hattam C. Pahl S. King H. Austen M. (2019). Are some natural environments more psychologically beneficial than others? The importance of type and quality on connectedness to nature and psychological restoration. Environ. Behav. 51, 111–143. doi: 10.1177/0013916517738312

[ref90] Yeo B. G. Takada H. Taylor H. Ito M. Hosoda J. Allinson M.. (2015). POPs monitoring in Australia and New Zealand using plastic resin pellets, and international pellet watch as a tool for education and raising public awareness on plastic debris and POPs. Mar. Pollut. Bull. 101, 137–145. doi: 10.1016/j.marpolbul.2015.11.006, PMID: 26586511

[ref91] Yoshida M. (2018). “Situation of municipal solid waste management in African cities-an interpretation of the information provided by the first ACCP meeting” in The Second Meeting of African Clean Cities Platform (ACCP) (Maputo, Mozambique: African Clean Cities Platform).

