# Supplementary material for: Impact of the citizen science project COLLECT on ocean literacy and well-being within a north/west African and south-east Asian context
Source: Front Psychol. 2023 Jun 14;14:1130596. doi: 10.3389/fpsyg.2023.1130596 (PMC10303996; doi:10.3389/fpsyg.2023.1130596)
Supplement: Supplementary file 1 [file Table_1.DOCX]

Supplementary Material

**Table of contents**

**Figures 1 – 6**: Perceived impacts of marine litter, at baseline, per country....................................2

**Figures 7 – 12**: Perceived causes of marine litter, at baseline, per country....................................5

**Table 1.** Attitudes towards beach litter removal at baseline, per country.......................................8

**Figures 13 – 18**: Self-reported litter-reducing behaviors at baseline, per country..........................9

**

*

*

**Supplementary Figure 1.** Perceived impacts of marine litter in Benin at pre-intervention (1 – 5 scale: *strongly disagree*–*strongly agree*). *Note.* Error bars represent standard error. **p* < .05; ***p* < .01.

**

*

**Supplementary Figure 2.** Perceived impacts of marine litter in Cabo Verde at pre-intervention (1 – 5 scale: *strongly disagree*–*strongly agree*). *Note.* Error bars represent standard error. **p* < .05; ***p* < .01.

**Supplementary Figure 3.** Perceived impacts of marine litter in Côte d’Ivoire at pre-intervention (1 – 5 scale: *strongly disagree*–*strongly agree*). *Note.* Error bars represent standard error.

**Supplementary Figure 4.** Perceived impacts of marine litter in Ghana at pre-intervention (1 – 5 scale: *strongly disagree*–*strongly agree*). *Note.* Error bars represent standard error.

*

**

**

**Supplementary Figure 5.** Perceived impacts of marine litter in Malaysia at pre-intervention (1 – 5 scale: *strongly disagree*–*strongly agree*). *Note.* Error bars represent standard error. **p* < .05; ***p* < .01.

*

*

*

**Supplementary Figure 6.** Perceived impacts of marine litter in Nigeria at pre-intervention (1 – 5 scale: *strongly disagree*–*strongly agree*). *Note.* Error bars represent standard error. **p* < .05.

**

**

**

**

**

*

**Supplementary Figure 7.** Perceived causes of marine litter in Benin at pre-intervention (1 – 5 scale: *strongly disagree*–*strongly agree*). *Note.* Error bars represent standard error. **p* < .05; ***p* < .01.

**

**

**

**

**Supplementary Figure 8.** Perceived causes of marine litter in Cabo Verde at pre-intervention (1 – 5 scale: *strongly disagree*–*strongly agree*). *Note.* Error bars represent standard error. ***p* < .01.

**

**

**

**Supplementary Figure 9.** Perceived causes of marine litter in Côte d’Ivoire at pre-intervention (1 – 5 scale: *strongly disagree*–*strongly agree*). *Note.* Error bars represent standard error. ***p* < .01.

**

**

**

*

**Supplementary Figure 10.** Perceived causes of marine litter in Ghana at pre-intervention (1 – 5 scale: *strongly disagree*–*strongly agree*). *Note.* Error bars represent standard error. **p* < .05; ***p* < .01.

*

**

**

**

**

*

**Supplementary Figure 11.** Perceived causes of marine litter in Malaysia at pre-intervention (1 – 5 scale: *strongly disagree*–*strongly agree*). *Note.* Error bars represent standard error. **p* < .05; ***p* < .01.

**

**

**

**

**Supplementary Figure 12.** Perceived causes of marine litter in Nigeria at pre-intervention (1 – 5 scale: *strongly disagree*–*strongly agree*). *Note.* Error bars represent standard error. ***p* < .01.

**Supplementary Table 1.** Attitudes towards beach litter removal at pre-intervention, per country (1 – 5 scale: *strongly disagree*–*strongly agree*). *Note.* **p* < .05; ***p* < .01.

|  | **Attitudes towards beach litter removal at baseline, per country** | **M (SD)** | **Significant differences** | |
| --- | --- | --- | --- | --- |
| 1  2  3  4  5 | **Benin**             Local government is responsible             Local community is responsible             Everyone is responsible, including me             Collective activities are important to keep the beach litter-free             Only the original polluters are responsible | 3.80 (0.99)  3.62 (0.96)  3.95 (1.22)  4.05 (0.82)  2.55 (1.27) | 1 > 5**  2 > 5*  3 > 5**  4 > 5** |  |
| 1  2  3  4  5 | **Cabo Verde**             Local government is responsible             Local community is responsible             Everyone is responsible, including me             Collective activities are important to keep the beach litter-free             Only the original polluters are responsible | 2.89 (0.96)  3.89 (0.76)  4.89 (0.32)  4.39 (0.78)  2.28 (0.96) | 3 > 1**  4 > 1*  2 > 5*  3 > 5**  4 > 5** |  |
| 1  2  3  4  5 | **Côte d’Ivoire**             Local government is responsible             Local community is responsible             Everyone is responsible, including me             Collective activities are important to keep the beach litter-free             Only the original polluters are responsible | 2.86 (1.12)  3.73 (1.32)  4.86 (0.35)  4.77 (0.53)  2.18 (1.40) | 3 > 1**  4 > 1**  3 > 5**  4 > 5** |  |
| 1  2  3  4  5 | **Ghana**             Local government is responsible             Local community is responsible             Everyone is responsible, including me             Collective activities are important to keep the beach litter-free             Only the original polluters are responsible | 2.70 (1.11)  3.81 (1.18)  4.72 (0.76)  4.39 (0.90)  1.87 (1.06) | 2 > 1**  3 > 1**  4 > 1** 3 > 2* | 2 > 5**  3 > 5**  4 > 5** |
| 1  2  3  4  5 | **Malaysia**  Local government is responsible             Local community is responsible             Everyone is responsible, including me             Collective activities are important to keep the beach litter-free             Only the original polluters are responsible | 3.64 (0.95)  3.88 (0.86)  4.24 (0.68)  4.14 (0.74)  3.05 (1.11) | 3 > 1*  2 > 5**  3 > 5**  4 > 5** |  |
| 1  2  3  4  5 | **Nigeria**            Local government is responsible             Local community is responsible             Everyone is responsible, including me             Collective activities are important to keep the beach litter-free             Only the original polluters are responsible | 3.17 (1.10)  3.53 (1.14)  4.47 (0.71)  4.35 (0.69)  2.19 (1.01) | 3 > 1**  4 > 1**  3 > 2**  4 > 2** | 1 > 5**  2 > 5**  3 > 5**  4 > 5** |

**

**

**

**

**

**Supplementary Figure 13.** Self-reported litter-reducing behaviors in Benin at pre-intervention (1 – 5 scale: *never*–*a great deal*). *Note.* Error bars represent standard error. ***p* < .01.

*

**

**Supplementary Figure 14.** Self-reported litter-reducing behaviors in Cabo Verde at pre-intervention (1 – 5 scale: *never*–*a great deal*). *Note.* Error bars represent standard error. **p* < .05; ***p* < .01.

**

**

**

*

**Supplementary Figure 15.** Self-reported litter-reducing behaviors in Côte d’Ivoire at pre-intervention (1 – 5 scale: *never*–*a great deal*). *Note.* Error bars represent standard error. **p* < .05; ***p* < .01.

**

**

**

**

**Supplementary Figure 16.** Self-reported litter-reducing behaviors in Ghana at pre-intervention (1 – 5 scale: *never*–*a great deal*). *Note.* Error bars represent standard error. ***p* < .01.

**

*

**

**

**

**

**Supplementary Figure 17.** Self-reported litter-reducing behaviors in Malaysia at pre-intervention (1 – 5 scale: *never*–*a great deal*). *Note.* Error bars represent standard error. **p* < .05; ***p* < .01.

**

**

**

**

**

**

**

**

**Supplementary Figure 18.** Self-reported litter-reducing behaviors in Nigeria at pre-intervention (1 – 5 scale: *never*–*a great deal*). *Note.* Error bars represent standard error. ***p* < .01.
